# Semaglutide Added to Basal Insulin in Type 2 Diabetes (SUSTAIN 5): A Randomized, Controlled Trial

**DOI:** 10.1210/jc.2018-00070

**Published:** 2018-04-23

**Authors:** Helena W Rodbard, Ildiko Lingvay, John Reed, Raymond de la Rosa, Ludger Rose, Danny Sugimoto, Eiichi Araki, Pei-Ling Chu, Nelun Wijayasinghe, Paul Norwood

**Affiliations:** 1 Endocrine and Metabolic Consultants, Rockville, Maryland; 2 Department of Internal Medicine, University of Texas Southwestern Medical Center, Dallas, Texas; 3 Endocrine Research Solutions, Inc., Roswell, Georgia; 4 Four Rivers Clinical Research, Paducah, Kentucky; 5 Institute of Diabetes Research, Münster, Germany; 6 Cedar-Crosse Research Center, Chicago, Illinois; 7 Department of Metabolic Medicine, Kumamoto University, Kumamoto, Japan; 8 Novo Nordisk Inc., Plainsboro, New Jersey; 9 Novo Nordisk A/S, Søborg, Denmark; 10 University of California at San Francisco, Fresno, California

## Abstract

**Context:**

Combination therapy with insulin and glucagon-like peptide-1 receptor agonists (GLP-1RAs) is important for treating type 2 diabetes (T2D). This trial assesses the efficacy and safety of semaglutide, a GLP-1RA, as an add-on to basal insulin.

**Objective:**

To demonstrate the superiority of semaglutide vs placebo on glycemic control as an add-on to basal insulin in patients with T2D.

**Design:**

Phase 3a, double-blind, placebo-controlled, 30-week trial.

**Setting:**

This study included 90 sites in five countries.

**Patients:**

We studied 397 patients with uncontrolled T2D receiving stable therapy with basal insulin with or without metformin.

**Interventions:**

Subcutaneous semaglutide 0.5 or 1.0 mg once weekly or volume-matched placebo.

**Main Outcome Measures:**

Primary endpoint was change in glycated Hb (HbA_1c_) from baseline to week 30. Confirmatory secondary endpoint was change in body weight from baseline to week 30.

**Results:**

At week 30, mean HbA_1c_ reductions [mean baseline value, 8.4% (67.9 mmol/mol)] with semaglutide 0.5 and 1.0 mg were 1.4% (15.8 mmol/mol) and 1.8% (20.2 mmol/mol) vs 0.1% (1.0 mmol/mol) with placebo [estimated treatment difference (ETD) vs placebo, –1.35 (14.8 mmol/mol); 95% CI, –1.61 to –1.10 and ETD, –1.75% (19.2 mmol/mol); 95% CI, –2.01 to –1.50; both *P* < 0.0001]. Severe or blood glucose–confirmed hypoglycemic episodes were reported in 11 patients (17 events) and 14 patients (25 events) with semaglutide 0.5 and 1.0 mg, respectively, vs seven patients (13 events) with placebo (estimated rate ratio vs placebo, 2.08; 95% CI, 0.67 to 6.51 and estimated rate ratio vs placebo, 2.41; 95% CI, 0.84 to 6.96 for 0.5 and 1.0 mg; both *P* = nonsignificant). Mean body weight decreased with semaglutide 0.5 and 1.0 mg vs placebo from baseline to end of treatment: 3.7, 6.4, and 1.4 kg (ETD, –2.31; 95% CI, –3.33 to –1.29 and ETD, –5.06; 95% CI, –6.08 to –4.04 kg; both *P* < 0.0001). Premature treatment discontinuation due to adverse events was higher for semaglutide 0.5 and 1.0 mg vs placebo (4.5%, 6.1%, and 0.8%), mainly due to gastrointestinal disorders.

**Conclusions:**

Semaglutide, added to basal insulin, significantly reduced HbA_1c_ and body weight in patients with uncontrolled T2D vs placebo.

## Teaser

The randomized, double-blind SUSTAIN 5 trial demonstrated the superiority of once-weekly semaglutide vs placebo in patients with uncontrolled T2D on basal insulin therapy.

Type 2 diabetes (T2D) is a complex disorder that requires individualized treatment strategies. Because of its progressive nature, many individuals receiving basal insulin require intensification of therapy to maintain optimal glycemic control and to reduce the risk of complications (1–5). Although increasing the basal insulin dose and/or adding mealtime insulin is often effective, this approach can increase the risk of hypoglycemia and lead to weight gain in an often overweight population (6).

Glucagon-like peptide-1 receptor agonists (GLP-1RAs) reduce blood glucose levels, with a low risk of hypoglycemia, and decrease body weight through reduced appetite and energy intake (7–9). Furthermore, GLP-1RAs in combination with basal insulin have been shown to reduce glycated Hb (HbA_1c_) and body weight, with a relatively low risk of hypoglycemia (10, 11). To improve treatment adherence and health-related quality of life for patients, recent efforts have focused on the development of once-weekly GLP-1RAs (12–14).

Semaglutide is a GLP-1 analog for the treatment of T2D. It has 94% amino acid sequence homology with native GLP-1 and is structurally similar to liraglutide (15, 16). Its minor structural modifications make it less susceptible to degradation by dipeptidyl peptidase-4 and improve binding to albumin (15). These modifications result in a half-life of ∼1 week (15), enabling once-weekly subcutaneous administration (17, 18).

The objective of this phase 3a, Semaglutide Unabated Sustainability in Treatment of Type 2 Diabetes (SUSTAIN) 5 trial was to demonstrate the superiority of once-weekly semaglutide (0.5 and 1.0 mg) vs placebo on glycemic control in patients with uncontrolled T2D on basal insulin therapy.

## Materials and Methods

### Trial design

This 30-week, randomized, double-blind, placebo-controlled, parallel-group, multinational, multicenter, four-armed trial (NCT02305381; Supplemental Fig. 1) recruited patients from 90 sites in Germany, Japan, Serbia, Slovakia, and the United States. The trial was conducted in compliance with the International Conference on Harmonization Good Clinical Practice guidelines (19) and the Declaration of Helsinki (20). The protocol, which is available online, was approved by the relevant institutional review boards.

### Patients

Eligible patients were ≥18 years of age (or ≥20 years of age in Japan) and diagnosed with T2D. All patients were receiving stable basal insulin therapy (minimum of 0.25 IU/kg/d and/or 20 IU/d of insulin glargine, insulin detemir, insulin degludec, and/or neutral protamine Hagedorn insulin) alone or in combination with metformin for 90 days prior to screening, with an HbA_1c_ level of 7.0% to 10.0% (53.0 to 85.8 mmol/mol). Key exclusion criteria included treatment with any glucose-lowering agent other than those listed herein in the 90 days prior to screening (excepting short-term bolus insulin therapy of ≤7 days); history of pancreatitis (acute or chronic); family history of medullary thyroid carcinoma or multiple endocrine neoplasia type 2; severe renal impairment (estimated glomerular filtration rate <30 mL/min/1.73 m^2^ according to the Modification of Diet in Renal Disease formula); more than three episodes of severe hypoglycemia within the 6 months prior to screening; known proliferative retinopathy or maculopathy requiring acute treatment; or being pregnant, breastfeeding, or intending to become pregnant. Full inclusion and exclusion criteria are listed in Supplemental Table 1. Written informed consent was obtained from all patients before trial-related activities commenced.

### Randomization and masking

Patients were randomized using an interactive voice/web-response system in a 2:2:1:1 ratio to receive once-weekly semaglutide (0.5 or 1.0 mg subcutaneously) or placebo administered subcutaneously as an add-on to pretrial background medication. Semaglutide and placebo treatments were, as far as possible, visually identical and packaged in a way so that study patients and investigators would not be able to distinguish between trial products. Furthermore, semaglutide and placebo were volume-matched during treatment to ensure blinding within dose level. The randomization was stratified according to HbA_1c_ level at screening (≤8.0% or >8.0%) and use of metformin (yes or no).

### Background medication and basal insulin titration

Patients with HbA_1c_ ≤8.0% (63.9 mmol/mol) at screening had their background basal insulin dose reduced by 20% at the start-of-trial medication to limit the potential risk of hypoglycemia. For these patients the insulin dose could be uptitrated from week 10 to week 16 (for further details, please see the titration protocol in the Supplemental Material). Increasing basal insulin dose before week 10 or after week 16 was avoided unless required to control acute hyperglycemia or prevent acute diabetic complications.

For all patients, insulin titrations were based on the lowest of three consecutive fasting self-measured blood glucose (SMBG) values according to a prespecified titration protocol (Supplemental Table 2). As far as possible, doses of basal insulin and metformin were to remain stable throughout the trial, with the exception of (1) dose reduction due to hypoglycemia or (2) confirmatory fasting plasma glucose (FPG) exceeding predefined limits per protocol, where the patient was offered intensification of therapy (rescue medication). Basal insulin dose increase was the first choice of rescue medication (Supplemental Material), which was initiated at the discretion of the investigator and in accordance with the American Diabetes Association (ADA)/European Association for the Study of Diabetes recommendations (21).

### Drug administration

Patients received semaglutide (0.5 or 1.0 mg subcutaneously) or volume-matched placebo once weekly for 30 weeks followed by a 5-week follow-up period. Study medication was administered following a fixed dose-escalation regimen. For 0.5 mg, the maintenance dose was reached after 4 weeks of 0.25 mg semaglutide or matching placebo once weekly. For 1.0 mg, the maintenance dose was reached after 4 weeks of 0.25 mg, followed by 4 weeks of 0.5 mg semaglutide or matching placebo once weekly. Trial products were manufactured and supplied by Novo Nordisk A/S (Bagsvaerd, Denmark).

### Outcomes

The primary outcome was the change in HbA_1c_ from baseline to week 30. The confirmatory secondary endpoint was the change in body weight from baseline to week 30. Secondary efficacy endpoints included the proportion of patients who achieved HbA_1c_ <7.0% (53 mmol/mol) (22) or ≤6.5% (48 mmol/mol) (23) by end of treatment; HbA_1c_ <7.0% without severe or blood glucose–confirmed symptomatic hypoglycemic episodes [plasma glucose level <3.1 mmol/L (56 mg/dL)] and no weight gain at week 30; change from baseline to week 30 in FPG; SMBG seven-point profiles and postprandial increments (mean over all meals); insulin dose; body mass index; waist circumference; proportion of patients who achieved weight loss of ≥5% and ≥10% at week 30; and change from baseline to week 30 of systolic and diastolic blood pressure, fasting lipids, and patient-reported outcomes [36-Item Short Form (SF-36v2) Health Survey and Diabetes Treatment Satisfaction Questionnaire].

Safety outcomes after 30 weeks of therapy included the number of treatment-emergent adverse events (AEs), severe or blood glucose–confirmed symptomatic hypoglycemic episodes (according to the ADA classification or blood glucose–confirmed by a plasma glucose value <3.1 mmol/L with symptoms consistent with hypoglycemia), and pulse rate. Hypoglycemia was assessed as a secondary safety endpoint. When a hypoglycemic episode was suspected, the blood glucose level at the time of the event was recorded as well as additional information relating to the circumstances of the patient. A hypoglycemic episode form was completed for each hypoglycemic episode; if the episode fulfilled the criteria for a serious AE, then an AE form and a safety information form were also completed. Hypoglycemic episodes were classified according to the Novo Nordisk A/S classification and ADA classification guidelines.

An external Event Adjudication Committee (EAC) validated predefined events of special interest [including those associated with GLP-1RA therapy: acute pancreatitis, neoplasm (excluding thyroid), and thyroid disease (including neoplasms)] in an independent, blinded manner (Supplemental Material).

### Statistical analysis

The trial was designed to establish superiority jointly for both doses of semaglutide vs pooled placebo (hereafter referred to as “placebo”) for the change in HbA_1c_ and body weight at week 30 with a one-sided *α* of 2.5%, assuming treatment differences vs placebo of 0.45% and 2.25 kg for each semaglutide dose level, and SDs of 1.1% and 4.0 kg. The type I error probability was controlled at 2.5% (one-sided) across the four confirmatory superior hypotheses in a hierarchical testing strategy. For HbA_1c_, the superiority of semaglutide vs placebo was tested first, starting with the highest semaglutide dose level, followed by body weight superiority vs placebo in the same dose order. Based on these assumptions and on a target sample size of 390 patients in total, the overall power to simultaneously demonstrate superiority on change in HbA_1c_ and body weight for the two dose levels of semaglutide vs placebo was 82% (full details are in Supplemental Material).

HbA_1c_, body weight, and other continuous endpoints assessed over time were analyzed using a mixed model for repeated measurements, with treatment, country, and stratification variables [HbA_1c_ level at screening (≤8.0% or >8.0%) crossed with use of metformin (yes or no)] as fixed factors and baseline value as covariate, all nested within visit (full details are in Supplemental Material). Efficacy evaluations were based on the full analysis set, comprising all randomized patients exposed to at least one dose of trial product. The primary analyses used data collected before initiation of any rescue medication or before premature treatment discontinuation. The robustness of the analyses of HbA_1c_ and body weight was assessed by handling missing data in various ways, including a placebo-based multiple imputation model, in which missing data points were imputed as if the patient was receiving placebo. Details of the sensitivity analyses are included in Supplemental Material.

## Results

### Patient disposition and baseline characteristics

In total, 397 patients were randomized to receive semaglutide or placebo from 1 December 2014 through 21 November 2015; 396 patients were exposed to treatment, and 380 patients completed the trial (Supplemental Fig. 2). A total of 23 patients required rescue medication: three in the semaglutide 0.5 mg group, one in the semaglutide 1.0 mg group, and 19 in the placebo group. Baseline characteristics were similar between treatment groups (Table 1). The mean duration of diabetes prior to trial entry was 13.3 years (range, 0.4 to 39.6 years). The majority of patients were receiving insulin glargine therapy at baseline (Table 1).

**Table 1.** Baseline Characteristics of the Study Population

	Semaglutide 0.5 mg (n = 132)	Semaglutide 1.0 mg (n = 131)	Placebo (n = 133)	Total (N = 396)
Male sex, n (%)	74 (56.1)	77 (58.8)	71 (53.4)	222 (56.1)
Country, n (%)				
Germany	25 (18.9)	24 (18.3)	21 (15.8)	70 (17.7)
Japan	17 (12.9)	22 (16.8)	22 (16.5)	61 (15.4)
Serbia	17 (12.9)	13 (9.9)	15 (11.3)	45 (11.4)
Slovakia	13 (9.8)	13 (9.9)	14 (10.5)	40 (10.1)
United States	60 (45.5)	59 (45.0)	61 (45.9)	180 (45.5)
Race, n (%)				
White	108 (81.8)	98 (74.8)	101 (75.9)	307 (77.5)
Asian	19 (14.4)	23 (17.6)	24 (18.0)	66 (16.7)
Black or African American	4 (3.0)	9 (6.9)	8 (6.0)	21 (5.3)
Other	0 (0.0)	1 (0.8)	0 (0.0)	1 (0.3)
Baseline HbA_1c_, n (%)*^a^*				
≤8.0% with metformin	41 (3.1)	41 (31.3)	40 (30.1)	122 (30.8)
≤8.0% without metformin	8 (6.1)	8 (6.1)	9 (6.8)	25 (6.3)
>8.0% with metformin	69 (52.3)	69 (52.7)	70 (52.6)	208 (52.5)
>8.0% without metformin	14 (10.6)	13 (9.9)	14 (10.5)	41 (10.4)
Age, mean (min.–max.), y	59.1 (28–84)	58.5 (33–80)	58.8 (19–86)	58.8 (19–86)
HbA_1c_, mean (min.–max.), mmol/mol*^b^*	67.9 (53.0–89.1)	67.3 (51.9–94.5)	68.6 (50.8–97.8)	67.9 (50.8–97.8)
HbA_1c_, mean (min.–max.), %*^b^*	8.4 (7.0–10.3)	8.3 (6.9–10.8)	8.4 (6.8–11.1)	8.4 (6.8–11.1)
Fasting plasma glucose, mean (min.–max.), mmol/L	8.9 (2.9–21.9)	8.5 (2.6–17.1)	8.6 (3.9–19.1)	8.6 (2.6–21.9)
Fasting plasma glucose, mean (min.–max.), mg/dL	161.0 (52.3–394.6)	152.5 (46.9–308.1)	154.1 (70.3–344.2)	155.9 (46.9–394.6)
Diabetes duration, mean (min.–max.), y	12.9 (0.4–37.1)	13.7 (0.6–36.9)	13.3 (0.8–39.6)	13.3 (0.4–39.6)
Body weight, mean (min.–max.), kg	92.7 (50.4–162.8)	92.5 (48.5–165.6)	89.9 (47.5–157.3)	91.7 (47.5–165.6)
Body mass index, mean (min.–max.), kg/m2	32.8 (21.1–51.4)	32.0 (19.5–51.6)	31.8 (21.0–48.8)	32.2 (19.5–51.6)
Basal insulin dose, mean (min.–max.), IU	39.3 (15.0–300.0)	37.4 (14.0–320.0)	36.6 (12.0–124.0)	37.7 (12.0–320.0)
Insulin glargine	42.6 (15.0–100.0)	50.3 (14.0–320.0)	43.4 (15.0–124.0)	45.4 (14.0–320.0)
Insulin detemir	56.1 (28.0–120.0)	40.1 (20.0–130.0)	40.0 (15.0–100.0)	44.3 (15.0–130.0)
Insulin degludec	63.8 (22.0–300.0)	30.3 (20.0–67.0)	35.5 (12.0–60.0)	39.8 (12.0–300.0)
NPH insulin	46.0 (20.0–130.0)	40.4 (20.0–80.0)	45.5 (20.0–124.0)	44.5 (20.0–130.0)
Oral diabetes medication, n (%)				
Metformin	110 (83.3)	110 (84.0)	110 (82.7)	330 (83.3)
Sulfonylureas	–	–	1 (0.8)*^c^*	1 (0.3)
Basal insulin at baseline, n (%)				
Insulin glargine	76 (57.6)	70 (53.4)	67 (50.4)	213 (53.8)
Insulin detemir	20 (15.2)	27 (20.6)	28 (21.1)	75 (18.9)
Insulin degludec	10 (7.6)	19 (14.5)	14 (10.5)	43 (10.9)
NPH insulin	27 (20.5)	15 (11.5)	24 (18.0)	66 (16.7)

Abbreviations: IU, international unit; max., maximum; min., minimum; NPH, neutral protamine Hagedorn; y, years.

^a^ Randomization was stratified according to HbA_1c_ level at screening (≤8.0% or >8.0%) and use of metformin (yes or no).

^b^ HbA_1c_ may be outside the range specified in the inclusion criteria because baseline measurement was conducted at randomization visit.

^c^ This patient was randomized in error and consequently excluded from the trial.

### Glycemic control

**Figure 1.**
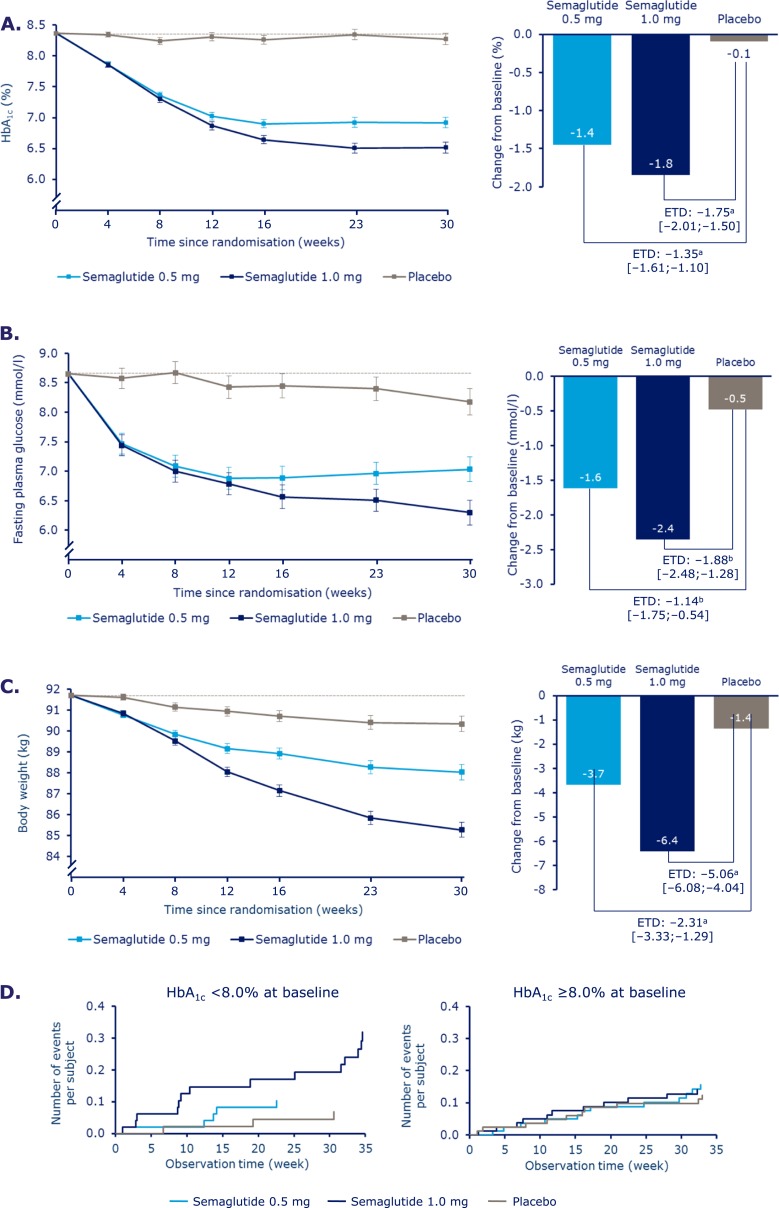
Change in (A) mean HbA_1c_, (B) fasting plasma glucose, (C) body weight, and (D) time to onset of hypoglycemia over time. Values are estimated means ± standard error from a mixed model for repeated measurements analysis using “on-treatment without rescue medication” data from patients in the full analysis set. The dashed line indicates the overall mean value at baseline. Values in square brackets indicate 95% CIs. ^a^Significant at *P* < 0.0001. ^b^Significant at *P* ≤ 0.0002. ETD, estimated treatment difference.

Mean HbA_1c_ [baseline 8.4%; SD, 0.83 (67.9 mmol/mol; SD, 9.04)] levels decreased over time (Fig. 1A; Supplemental Fig. 3A). At week 30, mean HbA_1c_ values with semaglutide 0.5 and 1.0 mg were 6.9% and 6.5%, vs 8.3% with placebo, corresponding to reductions of 1.4% and 1.8% vs 0.1% with placebo [estimated treatment difference (ETD) vs placebo, –1.35%; 95% CI, –1.61 to –1.10 and ETD, –1.75%; 95% CI, –2.01 to –1.50; both *P* < 0.0001]. The changes from baseline in HbA_1c_ in patients with HbA_1c_ ≤8% at screening were –0.88% (SD, 0.94), –1.32% (SD, 0.71), and –0.04% (SD, 0.98) with semaglutide 0.5 mg, semaglutide 1.0 mg, and placebo, respectively, whereas in patients with HbA_1c_ >8% the changes were –1.83% (SD, 1.00), –2.19% (SD, 0.86), and –0.28% (SD, 1.12), respectively.

**Figure 2.**
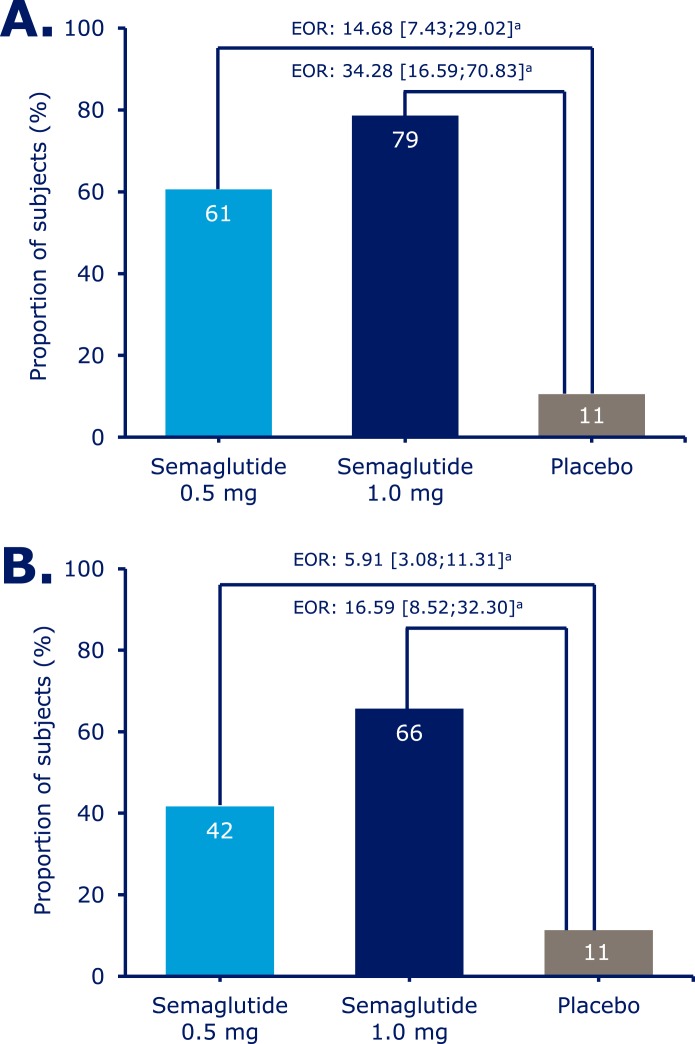
Patients achieving (A) an HbA_1c_ target of <7% and (B) a body weight loss target of ≥5% (American Association of Clinical Endocrinologists target) at week 30. Values are observed proportions using “on-treatment without rescue medication” data from patients in the full analysis set. Missing HbA_1c_ and body weight data are imputed from a mixed model for repeated measurements analysis and subsequently classified. Values in square brackets indicate 95% CIs. ^a^Significant at *P* < 0.0001. EOR, estimated OR.

**Figure 3.**
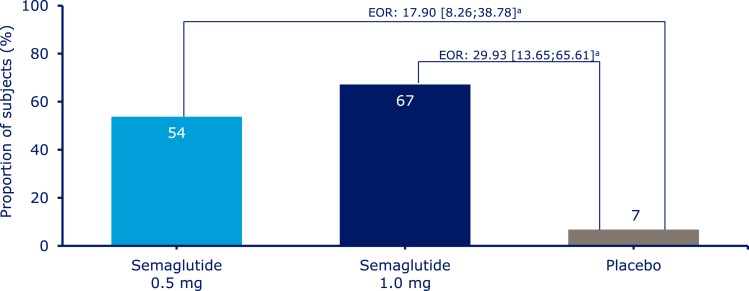
Patients achieving the composite endpoint target of HbA_1c_ <7.0% without severe or blood glucose–confirmed symptomatic hypoglycemia and with no weight gain. Values are observed proportions using “on-treatment without rescue medication” data from patients in the full analysis set. Missing HbA_1c_ and body weight data were imputed from a mixed model for repeated measurements analysis and subsequently classified. “Blood glucose-confirmed” defined as blood glucose <3.1 mmol/L (56 mg/dL). Values in square brackets indicate 95% CIs. ^a^Significant at *P* < 0.0001. EOR, estimated OR.

Significantly more patients achieved an HbA_1c_ target of <7.0% with semaglutide 0.5 mg and 1.0 mg than with placebo (61%, 79%, and 11%) (Fig. 2A) and an HbA_1c_ target ≤6.5% with semaglutide 0.5 and 1.0 mg vs placebo (41%, 61%, and 5%) (Supplemental Fig. 4A). Significantly more patients achieved a composite endpoint of <7.0% without severe or blood glucose–confirmed symptomatic hypoglycemia and with no weight gain with semaglutide 0.5 and 1.0 mg compared with placebo (54%, 67%, and 7%) (Fig. 3).

**Figure 4.**
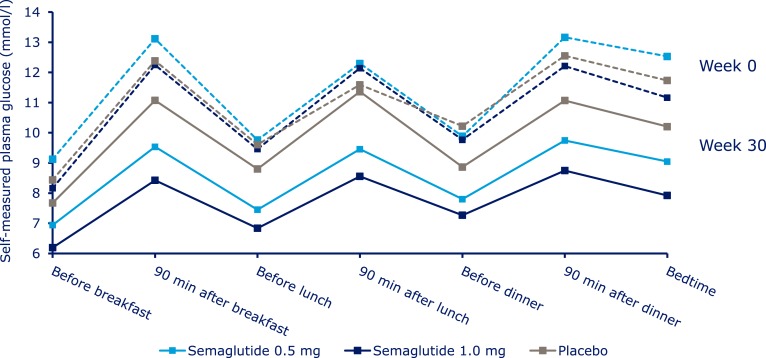
Observed mean seven-point self-measured blood glucose profile at baseline and at week 30. Values are observed means based on “on-treatment without rescue medication” data from patients in the full analysis set. Dashed lines represent week 0 data; solid lines represent week 30 data.

At week 30, mean FPG values with semaglutide 0.5 and 1.0 mg were 7.0 and 6.3 mmol/L, vs 8.2 mmol/L with placebo, corresponding to decreases of 1.6 and 2.4 mmol/L, compared with 0.5 mmol/L with placebo (ETD, –1.14; 95% CI, –17.5 to –0.54 and ETD, –1.88; 95% CI, –2.48 to –1.28; both *P* < 0.001) (Fig. 1B; Supplemental Table 3). Both incremental and mean seven-point SMBG decreased significantly with semaglutide 0.5 mg (by 0.8 and 2.5 mmol/L; both *P* < 0.004) and 1.0 mg (by 1.2 and 3.0 mmol/L; *P* < 0.0001 for both) vs placebo (reductions of 0.2 and 0.8 mmol/L) (Fig. 4; Supplemental Table 3).

Severe or blood glucose–confirmed hypoglycemic episodes were reported in 11 (8.3%) patients (17 events) in the semaglutide 0.5 mg group, in 14 (10.7%) patients (25 events) in the semaglutide 1.0 mg group, and in 7 (5.3%) patients (13 events) in the placebo group. The estimated rate ratio for events of severe or blood glucose–confirmed hypoglycemia was 2.08 (95% CI, 0.67 to 6.51; *P* = 0.2071) for semaglutide 0.5 mg vs placebo and 2.41 (95% CI, 0.84 to 6.96; *P* = 0.1030) for semaglutide 1.0 mg vs placebo. Among patients with HbA_1c_ ≤8.0% at screening, the proportion of patients reporting these events was higher in both semaglutide groups vs the placebo group and highest in the higher-dose semaglutide group (15.7, 46.5, and 9.9 events per 100 patient-years of exposure for semaglutide 0.5 mg, semaglutide 1.0 mg, and placebo, respectively). In subjects with HbA_1c_ >8%, the rates of “severe or BG-confirmed symptomatic” hypoglycemic episodes were comparable among the three groups (22.9, 21.2, and 18.6 events per 100 patient-years of exposure for semaglutide 0.5 mg, semaglutide 1.0 mg, and placebo, respectively) (Fig. 1D; Supplemental Table 5; Supplemental Fig. 8).

### Insulin dose

Insulin dose decreased from baseline to week 30 with semaglutide 0.5 mg, semaglutide 1.0 mg, and placebo (geometric means from 39.3 to 35.4, from 37.4 to 31.5, and from 36.6 to 35.2 IU). The end-of-treatment to baseline ratio for insulin dose with semaglutide 0.5 mg, semaglutide 1.0 mg, and placebo was 0.90, 0.85, and 0.96 (estimated treatment ratio for semaglutide 0.5 mg and 1.0 mg vs placebo, 0.94; 95% CI, 0.90 to 0.98 and estimated treatment ratio, 0.88; 95% CI, 0.84 to 0.92; *P* = 0.0046 and *P* < 0.0001). The largest overall decrease in insulin dose was in patients with baseline HbA_1c_ <8.0%. These patients reduced their insulin dose by 20% at randomization, in accordance with protocol (Supplemental Fig. 5).

### Body weight and related endpoints

At week 30, mean body weight decreased with semaglutide 0.5 and 1.0 mg vs placebo by 3.7, 6.4, and 1.4 kg (ETD for semaglutide 0.5 mg and 1.0 mg vs placebo, –2.31; 95% CI, –3.33 to –1.29 and ETD, –5.06; 95% CI, –6.08 to –4.04; both *P* < 0.0001) (Fig. 1C; Supplemental Fig. 3B). Body weight reductions of ≥5% were achieved in the semaglutide 0.5 mg, semaglutide 1.0 mg, and placebo groups by 42%, 66%, and 11% of patients and reductions of ≥10% by 9%, 26%, and 3% (Fig. 2B; Supplemental Fig. 4B).

### Other efficacy endpoints

Improvements in other efficacy endpoints, including lipid profile and systolic blood pressure, were observed with semaglutide vs placebo (Supplemental Tables 3 and 4). At week 30, mean systolic blood pressure values were 130.5 and 127.5 mm Hg for semaglutide 0.5 mg and 1.0 mg vs 133.8 mm Hg for placebo (ETD, –3.31; 95% CI, –6.92 to 0.31 and ETD, –6.29; 95% CI, –9.91 to –2.66; *P* = 0.0728 and *P* = 0.0007). Overall treatment satisfaction (measured by the Diabetes Treatment Satisfaction Questionnaire, status version) significantly improved with both semaglutide doses vs placebo (0.5 mg: ETD, 1.48; 95% CI, 0.14 to 2.82 and 1.0 mg: ETD, 2.22; 95% CI, 0.87 to 3.56) (Supplemental Fig. 6). No significant changes were evident from the SF-36v2 Health Survey questionnaire with either semaglutide dose compared with placebo.

### Safety and tolerability

The proportions of patients with AEs and serious AEs for semaglutide 0.5 and 1.0 mg vs placebo were 68.9%, 64.1%, and 57.9% and 6.1%, 9.2%, and 6.8% (Table 2). Rates of premature treatment discontinuation due to AEs were generally low but were higher with semaglutide 0.5 and 1.0 mg vs placebo (4.5% and 6.1% compared with 0.8% in the placebo group) and were primarily due to gastrointestinal (GI) AEs (Table 2).

**Table 2.** Adverse Events Overview

	Semaglutide 0.5 mg		Semaglutide 1.0 mg		Placebo	
	n (%)	Events (n)	n (%)	Events (n)	n (%)	Events (n)
No. of patients	132	–	131	–	133	–
AEs (total)	91 (68.9)	312	84 (64.1)	244	77 (57.9)	223
Fatal	0		0		0	
Serious	8 (6.1)	10	12 (9.2)	17	9 (6.8)	11
Severity*^a^*						
Severe	5 (3.8)	10	10 (7.6)	13	6 (4.5)	10
Moderate	42 (31.8)	84	32 (24.4)	57	28 (21.1)	55
Mild	81 (61.4)	218	68 (51.9)	174	64 (48.1)	158
Leading to premature treatment discontinuation	6 (4.5)	8	8 (6.1)	12	1 (0.8)	1
GI AEs						
Nausea	15 (11.4)	21	22 (16.8)	23	6 (4.5)	6
Vomiting	8 (6.1)	9	15 (11.5)	17	4 (3.0)	4
Diarrhea	6 (4.5)	6	9 (6.9)	9	2 (1.5)	2
Severe or BG-confirmed symptomatic hypoglycemia	11 (8.3)	17	14 (10.7)	25	7 (5.3)	13
Cardiovascular AEs	12 (9.1)	13	7 (5.3)	9	8 (6.0)	12
Gallbladder-related AEs	3 (2.3)	3	1 (0.8)	1	0	
EAC-confirmed neoplasms*^b^*	4 (3.0)	5	0		1 (0.8)	1
Benign	4 (3.0)	4	0		1 (0.8)	1
Colorectal	1 (0.8)	1	0		1 (0.8)	1
Skin	2 (1.5)	2	0		0	
Nasopharyngeal	1 (0.8)	1	0		0	
Malignant	1 (0.8)	1	0		0	
Skin	1 (0.8)	1	0		0	

Abbreviations: AE, adverse event; BG, blood glucose; EAC, Event Adjudication Committee; GI, gastrointestinal.

^a^ Severe, considerable interference with the subject's daily activities, unacceptable; moderate, marked symptoms, moderate interference with the subject's daily activities; mild, no or transient symptoms, no interference with the subject's daily activities.

^b^ There were five events in four patients with semaglutide 0.5 mg.

No fatalities were reported during the trial. One patient receiving semaglutide 0.5 mg became pregnant during the trial and discontinued treatment after 106 days (fetal exposure was ∼9 weeks). A healthy baby was born with no known congenital abnormalities.

The most frequent AEs with semaglutide were GI AEs (Table 2). Nausea was reported in 11.4% of patients treated with semaglutide 0.5 mg and in 16.8% treated with semaglutide 1.0 mg compared with 4.5% receiving placebo. In general, the prevalence of nausea events over time with semaglutide treatment was ∼3% to 5% throughout the study (Supplemental Fig. 7A). The proportions of patients reporting vomiting were 6.1%, 11.5%, and 3.0% (the prevalence of vomiting events over time is shown in Supplemental Fig. 7B), and the proportions reporting diarrhea were 4.5%, 6.9%, and 1.5%. All GI AEs were mild to moderate in severity. Pulse rate increased from baseline by 1 beat per minute (bpm) with semaglutide 0.5 mg and by 4 bpm with semaglutide 1.0 mg, vs a decrease of 1 bpm with placebo (for semaglutide 1.0 mg vs placebo, *P* < 0.0001; Supplemental Table 3). The proportion of patients with diabetic retinopathy (DR) events was 3.0% with semaglutide 0.5 mg and 0.8% with semaglutide 1.0 mg; there were no patients with DR events on placebo.

Gallbladder-related AEs were reported in four semaglutide-treated patients. With semaglutide 0.5 mg, cholelithiasis, gallbladder disorder, and blood alkaline phosphatase were reported in three patients; with semaglutide 1.0 mg, acute cholecystitis was reported in one patient (Table 2). Mean lipase and amylase activity increased significantly from baseline to end of treatment with both semaglutide doses compared with placebo (*P* < 0.0001 for all) (Supplemental Table 6; Supplemental Fig. 9), although no pancreatitis events were confirmed by the EAC. Two investigator-reported events were sent for adjudication: one patient (female, age 47 years) receiving semaglutide 0.5 mg reported acute pancreatitis, which led to premature treatment discontinuation (reported on study day 152, concurrent mild vomiting); the second patient (male, age 55 years) receiving semaglutide 1.0 mg reported elevated lipase but had an elevated level prior to administration of trial product (reported on study day 1, concurrent mild vomiting).

Neoplasms were confirmed by the EAC in four patients treated with semaglutide 0.5 mg and in one patient receiving placebo (Table 2). One malignant neoplasm (basal cell carcinoma) was reported in a patient treated with semaglutide 1.0 mg. Additionally, one patient treated with semaglutide 1.0 mg had confirmed metastatic pancreatic cancer with an onset date of 65 days after the end of treatment. There were no confirmed thyroid malignancies.

## Discussion

In patients with T2D inadequately controlled with basal insulin, semaglutide provided superior improvements in mean HbA_1c_, FPG, and SMBG and superior weight loss compared with placebo. There was a low rate of hypoglycemic episodes across the trial. Compared with placebo, severe or blood glucose–confirmed symptomatic episodes were more frequent with semaglutide in patients with HbA_1c_ ≤8.0% at screening, which might be expected given the improvements in HbA_1c_ in these patients vs placebo. A greater pre-emptive insulin dose reduction, greater than the 20% mandated by the protocol in this trial, may be appropriate when titrating from 0.5 to 1.0 mg in clinical practice.

In this study population with a mean diabetes duration of >13 years, the majority of patients in both semaglutide-treatment groups attained an HbA_1c_ target of <7.0%. In addition, significant weight loss was observed with both doses of semaglutide vs placebo. The improvements (weight reductions of 2.3 to 5.1 kg) with semaglutide appear to be greater in magnitude than those previously reported for other long-acting GLP-1RAs added to basal insulin (weight reductions of 1.0 to 1.6 kg), although these are cross-trial observations and therefore are not directly comparable (8, 24–29).

A significantly larger proportion of patients receiving semaglutide achieved the composite endpoint of HbA_1c_ <7.0% without severe or blood glucose–confirmed symptomatic hypoglycemia (plasma glucose level below 3.1 mmol/L) and without weight gain compared with placebo. This finding is clinically meaningful and especially promising in a population where hypoglycemia and weight gain impede insulin titration and glycemic control.

Semaglutide was well tolerated in this trial, with an AE profile similar to that of other GLP-1RAs. GI symptoms were the most common AEs with semaglutide and were responsible for the higher proportion of patients discontinuing treatment prematurely compared with placebo. This effect has been noted previously with GLP-1RAs, and GI AEs commonly occur early in the course of treatment and subside over time (30). Accordingly, in this study, nausea events in the semaglutide groups occurred mainly in the first 8 to 9 weeks of treatment, generally coinciding with the dose-escalation steps, and declined gradually thereafter until the end of the trial. Although the proportion of patients experiencing an event at least once was ∼11% to 17% with semaglutide, the actual prevalence was ∼3% to 5% on any given week. The prevalence of vomiting over time remained ≤2.5% in both semaglutide groups, and the majority of events occurred before week 18 of the study.

Similarly, the significant increase in pulse rate observed with semaglutide 1.0 mg compared with placebo has been previously reported in patients treated with GLP-1RAs (31, 32); however, the reasons for this hemodynamic effect are unclear (33). DR AEs in SUSTAIN 5 are discussed in a publication that analyzes DR in the SUSTAIN clinical program. An analysis of DR AEs in SUSTAIN 1–5 and the Japanese trials showed no evidence of increased DR AEs with semaglutide vs placebo or active comparators (34). One malignant neoplasm (basal cell carcinoma) was reported in the study in a patient treated with semaglutide 1.0 mg. In the same treatment group, one patient had metastatic pancreatic cancer, although the recorded date of onset was 65 days after the end of treatment. In published data from the SUSTAIN program, the overall incidence of malignant neoplasms was similarly low, with no evident imbalances between semaglutide dose groups or between semaglutide and comparators (35–37). No pancreatitis events were confirmed by the EAC, a finding that aligns with a recent meta-analysis of incretin-based medications and the reported risk of such events (38). In the analysis, which included results from the SUSTAIN 6 trial (39), the relative risk of both acute pancreatitis and pancreatic carcinoma was reduced for semaglutide and the GLP-1RA class overall (38).

Semaglutide treatment was associated with improvements in treatment satisfaction compared with placebo. However, despite the double-blind study design, the substantial differences in efficacy (*e.g.*, weight loss) and safety (*e.g.*, GI AEs) between semaglutide and placebo may have unblinded the study in many patients by week 30, thereby affecting the patient’s evaluation of the treatment. Nevertheless, therapies demonstrating increased treatment satisfaction in T2D could be beneficial, particularly because insulin therapy for diabetes is associated with a high treatment burden and compliance issues (40).

The strengths of this study include its randomized, double-blind, placebo-controlled design, which adds validity to the evaluation of outcomes. The trial was conducted in patients with long-standing T2D who were receiving basal insulin, and, although the majority was receiving insulin glargine therapy at baseline, a diverse range of insulin types was used, reflecting the international nature of the trial sites in this study.

This was not a treat-to-target study; thus, titration was carried out at the investigators’ discretion, with the exception of the mandatory dose decrease in patients with HbA_1c_ ≤8%. The lack of dose uptitration in the placebo arm may have been partially responsible for HbA_1c_ and FPG levels remaining elevated in this treatment group. Furthermore, because basal insulin was not titrated to target and because the placebo arm had no active intervention, the comparison of hypoglycemia rates between the arms and the applicability of the study findings to clinical practice should be interpreted according to these limitations.

The relatively short study length was a limitation, and the blinding aspect of the trial may have been partially lost due to the incidence of GI AEs and the improved fasting glucose levels over placebo. Because of the reduced insulin dose in the cohort with HbA_1c_ ≤8% and the subsequent delay before uptitration could be commenced, the results relating to weight loss and reduced insulin requirements should be interpreted in this context. In addition, the observed decrease in insulin dose and in body weight in the placebo group was unexpected, and the reasons for these anomalous effects are unclear.

In summary, semaglutide, administered subcutaneously once weekly, provided superior glycemic control and body weight reductions compared with placebo in patients with T2D receiving basal insulin therapy. No unexpected safety issues were identified. Semaglutide was well tolerated, with a safety profile similar to that of other GLP-1RAs.

## Supplementary Material

Supplemental Data

## Abbreviations:

ADA: American Diabetes Association
AE: adverse event
bpm: beats per minute
DR: diabetic retinopathy
EAC: Event Adjudication Committee
ETD: estimated treatment difference
FPG: fasting plasma glucose
GI: gastrointestinal
GLP-1RA: glucagon-like peptide-1 receptor agonist
HbA_1c_: glycated Hb
SF-36v2: 36-Item Short Form
SMBG: self-measured blood glucose
SUSTAIN: Semaglutide Unabated Sustainability in Treatment of Type 2 Diabetes
T2D: type 2 diabetes
