# Supplementary material for: Semaglutide Added to Basal Insulin in Type 2 Diabetes (SUSTAIN 5): A Randomized, Controlled Trial
Source: J Clin Endocrinol Metab. 2018 Apr 23;103(6):2291–301. doi: 10.1210/jc.2018-00070 (PMC5991220; doi:10.1210/jc.2018-00070)
Supplement: Supplemental Data [file jc.2018-00070.sd1.docx]

# SUSTAIN 5: Supplement

# Contents page

|  | | Page |
| --- | --- | --- |
| SUSTAIN 5 Committee Members and Investigators | | 2 |
| 1. Rescue criteria and medication | | 11 |
| 2. Events adjudicated by the external Event Adjudication Committee | | 11 |
| 3. Details of statistical analysis | | 11 |
| 4. The sensitivity of the primary and confirmatory secondary endpoint was assessed in four separate sensitivity analyses | | 12 |
| Figure S1 | Trial design | 13 |
| Figure S2 | Subject disposition (CONSORT) | 14 |
| Figure S3 | Sensitivity analyses | 15 |
| Figure S4 | Subjects achieving (A) a HbA_1c_ target of ≤6.5% (American Association of Clinical Endocrinologists target) and (B) a body weight loss target of ≥10% at Week 30 | 16 |
| Figure S5 | Insulin dose: observed ratio to baseline by week and HbA_1c_ at screening | 17 |
| Figure S6 | Subject-reported outcomes: change in Diabetes Treatment Satisfaction Questionnaire(s) compared with placebo for semaglutide 0.5 mg and semaglutide 1.0 mg | 18 |
| Figure S7 | Prevalence of (A) nausea events and (B) vomiting events | 19 |
| Figure S8 | Time to onset of first severe or blood glucose-confirmed symptomatic hypoglycemic event | 20 |
| Figure S9 | Lipase (A) and amylase (B) estimated mean levels by week and ratio to baseline at Week 30 | 21 |
| Table S1 | Inclusion and exclusion criteria | 22 |
| Table S2 | Insulin titration during the study | 23 |
| Table S3 | Change from baseline to Week 30 in primary and secondary efficacy outcomes by treatment group | 24 |
| Table S4 | Change from baseline to Week 30 for secondary endpoints | 25 |
| Table S5 | Hypoglycemic episodes | 27 |
| Table S6 | Amylase and lipase at Week 30 by treatment group | 28 |

## SUSTAIN 5 Committee Members and Investigators

**Cardiologists who reviewed the interpretation of the ECGs:** Rajdeep Agrawal, Quintiles Cardiac Safety Services, Mumbai, India; Shivakumar Bhairappa, Quintiles Cardiac Safety Services, Bangalore, India; Vijay Chowdekar, Quintiles Cardiac Safety Services, Bangalore, India; Anil Damle, Quintiles Cardiac Safety Services, Mumbai, India; Bhupen Desai, Quintiles Cardiac Safety Services, Mumbai, India; Satish Karur, Quintiles Cardiac Safety Services, Bangalore, India; Hema Kulkarni, Quintiles Cardiac Safety Services, Mumbai, India; Divyaprakash Madaiah, Quintiles Cardiac Safety Services, Bangalore, India; Ravi Math, Quintiles Cardiac Safety Services, Bangalore, India; Shivanand S Patil, Quintiles Cardiac Safety Services, Bangalore, India; Dattareya Rao, Quintiles Cardiac Safety Services, Bangalore, India; Subramani K. S., Quintiles Cardiac Safety Services, Bangalore, India.

**Part of the calcitonin monitoring committee and endocrinologist reviewers:** Laszlo Hegedüs, Department of Endocrinology and Metabolism, Odense University Hospital, Odense M, Denmark; Steven L. Sheerman, Department of Endocrine Neoplasia and Hormonal Disorders, The University of Texas MD Anderson Cancer Center, Houston, TX, USA; Robert Michael Tuttle, Memorial Sloan-Kettering Cancer Center and Weill, Medical College of Cornell University, Brewster, NY, USA.

**Central reviewer of pathology slides:** Roland A. Ghossein, Memorial Sloan Kettering Cancer Center, New York, NY, USA.

**Part of the data monitoring committee, an external committee of experts who independently reviewed and evaluated accumulating safety data at predefined time points using unblinded data:** Maria L. Aguilar, Mayo Clinic, Phoenix, AZ, USA; Levent Akduman, Saint Louis University Eye Institute, Department of Ophthalmology, Saint Louis University, St. Louis, MO, USA; Stewart G. Albert, St. Louis University School of Medicine, Division of Endocrinology, St. Louis, MO, USA; Amer Alshekhlee, Interventional Vascular Neurologist, SSM Neuroscience, Institute, DePaul Health Center, St. Louis, MO, USA; Richard Gordon Bach, Washington University School of Medicine, Cardiology Division, St. Louis, MO, USA; Judith D. Bebchuk, Puma BioTechnology, CA, USA; Therese M. Bertram, Independent consultant, Valmeyer, IL, USA; Geoffrey Allan Block, Denver Nephrologist Research Division, Denver Nephrologist, PC, Denver, CO, USA; Nicholas Boon, Royal Infirmary of Edinburgh, United Kingdom; David A Bushinsky, University of Rochester Medical Center, Nephrology, Division, Rochester, NY, USA; Bernard R. Chaitman, St. Louis University Core ECG Laboratory, St. Louis, MO, USA; Salvador Cruz-Flores, Sidney W. Souers Endowed Chair, Department of Neurology & Psychiatry, Chesterfield, MO, USA; Blonie Wayne Dudney, National Retina Institute, Baltimore, Retina Associates of St. Louis, Inc, Florissant, MO, USA; Eliahu Solomon Feen, Saint Louis University School of Medicine, Department of Neurology and Psychiatry, St. Louis, MO, USA; David Fitchett, St Michael’s Hospital, Toronto, Canada; Timothy Gardner, Dartmouth-Hitchcock Medical Center; Lebanon, USA; Gilbert Gosselin, Institute de Cardiologie de Montréal, Montréal, Canada; Chandra Prakash Gyawali, Washington University School of Medicine, St. Louis, MO, USA; Silvio Inzucchi, Yale University School of Medicine, USA; Meagan Anne Jacoby, Department of Medicine, Division of Oncology, Section of Stem Cell Biology, University City, MO, USA; Abhay Atul Laddu, Saint Louis University Medical Center, St. Louis, MO, USA; Kevin J. Martin, Saint Louis University Medical Center, Division of Nephrology, St. Louis, MO, USA; Daniel Kast Mullady, Washington University School of Medicine, Division of Gastroenterology, St. Louis, MO, USA; Michael J. Naughton, Washington University School of Medicine, St. Louis, MO, USA; Joel Picus, Washington University School of Medicine, St. Louis, MO, USA; Geoffrey L. Uy, Division of Oncology, Washington University School of Medicine, St. Louis, MO, USA.

**Study Investigators**

Germany: Ulrich Aigner, VERSDIAS GmbH, Sulzbach-Rosenberg; Anna Bartnik-Mikuta, VERSDIAS GmbH, Sulzbach-Rosenberg; Desirée Braun, Praxis Segner-Dr.Braun-Kirsch, St. Ingbert; Ulrich Clever, Diabetespraxis Hamburg-Blankenese, Hamburg; Dominik Dahl, Gemeinschaftspraxis für Innere Medizin, Hamburg; Ralf-Jörg Denger, Gemeinschaftspraxis, Friedrichsthal; Rudolf Erlinger, Medizinischen Zentrum Mönchfeld, Stuttgart; Moritz Erlinger, Medizinischen Zentrum Mönchfeld, Stuttgart; Michael Esser, Praxis Dr. med. Michael Esser, Essen; Katharina Gaede, Gemeinschaftspraxis für Innere Medizin, Hamburg; Adam Kilimnik, Schwerpunktpraxis für Diabetes, Gefäß- & Ernährungsmedizin Dr. Lüdemann , Falkensee; Andreas Klinge, Gemeinschaftspraxis für Innere Medizin, Hamburg; Katrin Krause, Hausarzt und Diabetologische Schwerpunktpraxis Dr Karsten Milek, Hohenmölsen; Benedict Lacner, Praxis Dr. med. Michael Esser, Essen; Dirk Lammers, Institut für Diabetesforschung GmbH in Münster im FranziskusCarré, Münster; Jörg Lüdeman, Schwerpunktpraxis für Diabetes, Gefäß- & Ernährungsmedizin Dr. Lüdemann , Falkensee; Hans-Peter Lüdemann, Schwerpunktpraxis für Diabetes, Gefäß- & Ernährungsmedizin Dr. Lüdemann , Falkensee; Karsten Milek, Hausarzt und Diabetologische Schwerpunktpraxis Dr Karsten Milek, Hohenmölsen; Björn Paschen, Diabetologische Schwerpunktpraxis Harburg, Hamburg; Harald Pohlmeier, Institut für Diabetesforschung GmbH in Münster im FranziskusCarré, Münster; Ludger Rose, Institut für Diabetesforschung GmbH in Münster im FranziskusCarré, Münster; Armin Sammler, Gemeinschaftspraxis, Friedrichsthal; Daniel Schlott, Diabetespraxis Hamburg-Blankenese, Hamburg; Bernd-M. Scholz, Diabetologische Schwerpunktpraxis Harburg, Hamburg; Alexander Segner, Praxis Segner-Dr.Braun-Kirsch, St. Ingbert; Ulrich Wendisch, Gemeinschaftspraxis für Innere Medizin, Hamburg; Josef Wenzl, Dr.V.Wenzl-Bauer Dr.J.Wenzl - Praxis für Diabetologie & Allgemeinmedizin, Rehlingen-Siersburg; Veronika Wenzl-Bauer, Dr.V.Wenzl-Bauer Dr.J.Wenzl - Praxis für Diabetologie & Allgemeinmedizin, Rehlingen-Siersburg.

Japan: Reiko Adachi, Takatsuki Red Cross Hospital, Osaka; Kazushi Amano, Heiwadai Hospital, Miyazaki; Tomohisa Aoyama, Tokyo-Eki Center-building Clinic, Tokyo; Eiichi Araki, Kumamoto University Hospital, Kumamoto; Shiho Fukai, Tokyo-Eki Center-building Clinic, Tokyo; Yasushi Fukushima, Tokyo-Eki Center-building Clinic, Tokyo; Noboru Furukawa, Kumamoto University Hospital, Kumamoto; Nobuaki Hirota, Tokyo-Eki Center-building Clinic, Tokyo; Tetsuya Homma, Tokyo-Eki Center-building Clinic, Tokyo; Hiromitsu Hotta, Tokyo-Eki Center-building Clinic, Tokyo; Motoyuki Igata, Kumamoto University Hospital, Kumamoto; Yuko Imada, Takatsuki Red Cross Hospital, Osaka; Hidenori Ishida, Nakakinen Clinic , Ibaraki; Norio Ishii, Kumamoto University Hospital, Kumamoto; Yorihiro Iwasaki, Takatsuki Red Cross Hospital, Osaka; Shinsuke Iwashita, Kumamoto University Hospital, Kumamoto; Masashi Izumiya, Tokyo-Eki Center-building Clinic, Tokyo; Sunao Kaneko, Tokyo-Eki Center-building Clinic, Tokyo; Shizuka Kaneko, Takatsuki Red Cross Hospital, Osaka; Shuji Kawasaki, Kumamoto University Hospital, Kumamoto; Junji Kawashima, Kumamoto University Hospital, Kumamoto; Takako Kikuchi, Tokyo-Eki Center-building Clinic, Tokyo; Hiroyuki Kinoshita, Kumamoto University Hospital, Kumamoto; Arihiro Kiyosue, Tokyo-Eki Center-building Clinic, Tokyo; Tatsuya Kondo, Kumamoto University Hospital, Kumamoto; Daisuke Kukidome, Kumamoto University Hospital, Kumamoto; Yoshifumi Maeno, Shiraiwa Clinic, Osaka; Takeshi Matsumura, Kumamoto University Hospital, Kumamoto; Hiroko Mizuma, Tokyo-Eki Center-building Clinic, Tokyo; Ryo Morimoto, Tokyo-Eki Center-building Clinic, Tokyo; Hiroyuki Motoshima, Kumamoto University Hospital, Kumamoto; Shuji Nakamura, Heiwadai Hospital, Miyazaki; Koujin Nakayama, Heiwadai Hospital, Miyazaki; Takeshi Nishikawa, Kumamoto University Hospital, Kumamoto; Hiroko Nishioka, Kumamoto University Hospital, Kumamoto; Satoshi Noguchi, Tokyo-Eki Center-building Clinic, Tokyo; Takafumi Oga, Tokyo-Eki Center-building Clinic, Tokyo; Takeshi Osonoi, Nakakinen Clinic , Ibaraki; Yusuke Osonoi, Nakakinen Clinic , Ibaraki; Aya Osonoi, Nakakinen Clinic , Ibaraki; Kazue Ota, Tokyo-Eki Center-building Clinic, Tokyo; Miki Ota, Tokyo-Eki Center-building Clinic, Tokyo; Miyoko Saito, Nakakinen Clinic , Ibaraki; Takafumi Senokuchi, Kumamoto University Hospital, Kumamoto; Seiya Shimoda, Kumamoto University Hospital, Kumamoto; Toshihiko Shiraiwa, Shiraiwa Clinic, Osaka; Yuka Shiraiwa, Shiraiwa Clinic, Osaka; Hidekazu Suzuki, Tokyo-Eki Center-building Clinic, Tokyo; Yumiko Tahara, Takatsuki Red Cross Hospital, Osaka; Shotaro Taniguchi, Heiwadai Hospital, Miyazaki; Atsuko Tamasawa, Nakakinen Clinic , Ibaraki; Megumi Tarui, Tokyo-Eki Center-building Clinic, Tokyo; Yohei Ueda, Takatsuki Red Cross Hospital, Osaka; Hiroshi Umadome, Takatsuki Red Cross Hospital, Osaka; Yuko Yamada, Shiraiwa Clinic, Osaka; Kaoru Yamamoto, Shiraiwa Clinic, Osaka; Takeshi Yamamotoya, Tokyo-Eki Center-building Clinic, Tokyo.

Serbia: Dragana Bubanja, Clinical Centre Kragujevac, Internal Diseases Clinic, Endocrinology Centre, Kragujevac; Aleksandar Djukic, Clinical Centre Kragujevac, Internal Diseases Clinic, Endocrinology Centre, Kragujevac; Zorana Djuran, Military Medical Academy, Clinic for Endocrinology, Belgrade; Tamara Dragovic, Military Medical Academy, Clinic for Endocrinology, Belgrade; Zoran Hajdukovic, Military Medical Academy, Clinic for Endocrinology, Belgrade; Tijana Icin, Clinical Centre Vojvodina, Clinic for Endocrinology, Diabetes and Metabolic Diseases, Novi Sad; Snezana Kuzmic Jankovic, Military Medical Academy, Clinic for Endocrinology, Belgrade; Jelena Karajovic, Military Medical Academy, Clinic for Endocrinology, Belgrade; Sasa Kikovic, Military Medical Academy, Clinic for Endocrinology, Belgrade; Milena Mitrovic, Clinical Centre Vojvodina, Clinic for Endocrinology, Diabetes and Metabolic Diseases, Novi Sad; Violeta Mladenovic, Clinical Centre Kragujevac, Internal Diseases Clinic, Endocrinology Centre, Kragujevac; Dragana Tomic Naglic, Clinical Centre Vojvodina, Clinic for Endocrinology, Diabetes and Metabolic Diseases, Novi Sad; Jovanka Novakovic Paro, Clinical Centre Vojvodina, Clinic for Endocrinology, Diabetes and Metabolic Diseases, Novi Sad; Jelena Petrovic, Clinical Centre Kragujevac, Internal Diseases Clinic, Endocrinology Centre, Kragujevac; Djordje Popovic, Clinical Centre Vojvodina, Clinic for Endocrinology, Diabetes and Metabolic Diseases, Novi Sad; Petar Ristic, Military Medical Academy, Clinic for Endocrinology, Belgrade; Edita Stokic, Clinical Centre Vojvodina, Clinic for Endocrinology, Diabetes and Metabolic Diseases, Novi Sad; Ivan Tavcar, Military Medical Academy, Clinic for Endocrinology, Belgrade; Bojan Vukovic, Clinical Centre Vojvodina, Clinic for Endocrinology, Diabetes and Metabolic Diseases, Novi Sad; Branka Kovacev Zavisic, Clinical Centre Vojvodina, Clinic for Endocrinology, Diabetes and Metabolic Diseases, Novi Sad.

Slovakia: Jan Antolik, DIA – KONTROL s.r.o, Ambulancia diabetologie poruchy latkovej vymeny a vyzivy, Levice; Katarina Cerna, UNB Bratislava, Nemocnica akademika L. Derera, Diabetologicka ambulancia, Bratislava; Viera Donicova, HUMAN-CARE s.r.o., Interna a Diabetologicka Ambulancia, Kosice; Marek Macko, DIABETOL s.r.o., Diabetologicka ambulancia, Presov; Livia Tomasova, IN-DIA s.r.o., Diabetologicka ambulancia, Lucenec; Patricia Tomasova, IN-DIA s.r.o., Diabetologicka ambulancia, Lucenec; Anna Vargova, DIA – KONTROL s.r.o, Ambulancia diabetologie poruchy latkovej vymeny a vyzivy, Levice.

United States: Marconi Abreu, UT Southwestern Medical Center at Dallas, Dallas, TX; John Agaiby, Clinical Investigation Specialists, Inc., Kenosha, WI; Eva Agaiby, Clinical Investigation Specialists, Inc., Kenosha, WI; Rachid Alaoui, Carl R. Meisner Medical Clinic, PLLC, Sugarland, TX; John Edward Albers, CTI Clinical Research Center, Crestview Hills, KY; Kurt Alexander, Community Clinical Research, Muncie, IN; Muhammad Luay Alkotob, Elite Research Center, Flint, MI; Eunice Allen, Galenos Research, Dallas, TX; Mohammand Al-Raje, Albuquerque Clinical Trials, Inc., Albuquerque, NM; Janet Andazola, Infosphere Clinical Studies, Inc., West Hills, CA; James Anderson, Meridien Research, Brooksville, FL; Lucas Anderson, Valley Research, Fresno, CA; Michael Anderton, Clinical Research Advantage/ Family Practice Specialists, Phoenix, AZ; Alisha Kristine Riggs Archibeck, Clinical Research Advantage/ Family Practice Specialists, Phoenix, AZ; Priscilla Armijo, Albuquerque Clinical Trials, Inc., Albuquerque, NM; Claudia Ortiz Asberry, East Coast Institute for Research, LLC, Fleming Island , FL; Cheryl Auston, North Shore University Health System, Skokie, IL; Vanessa Avila, Clinical Research Advantage/ Family Practice Specialists, Phoenix, AZ; Marcela Vargas Avila, National Research Institute, Los Angeles, CA; Lianne Azar, Meridien Research, Brooksville, FL; Masoud Azizad, Valley Clinical Trials, Northridge, CA; Trista Bachand, Diabetes and Thyroid Center of Fort Worth, Fort Worth , TX; David Badham, Wade Family Medicine, Bountiful, UT; Antoinette Bailey, East Coast Institute for Research, LLC, Fleming Island , FL; Chris Bajaj, Diabetes and Thyroid Center of Fort Worth, Fort Worth , TX; Muhammad Ayman Bakleh, Elite Research Center, Flint, MI; George Barchini, Chase Medical Research LLC, Waterbury, CT; Heather Barnes, INTEGRIS Family Care Yukon, Yukon, OK; Brett Baxter, Clinical Research Advantage/ Family Practice Specialists, Phoenix, AZ; Donna Beheyt, The Center for Sexual Health, Metairie, LA; Brenda Benavidez, Briggs Clinical Research, LLC, San Antonio, TX; Mark Benson, American Health Network of Indiana, LLC, Avon, IN; Vivian Rita Berger, Sterling Research Group, Ltd., Cincinnati, OH; Holly (Baxter) Berryman, Four Rivers Clinical Research Inc., Paducah, KY; Martin Bialow, Meridien Research, Tampa, FL; William Biggs, Amarillo Medical Specialists, Amarillo, TX; Alistaire Bilas, Anaheim Clinical Trials, LLC, Anaheim, CA; Liana Billings, North Shore University Health System, Skokie, IL; Herman Blomeier, North Shore University Health System, Skokie, IL; Bryon Boley, Westside Family Medical Center, P.C., Kalamazoo, MI; Charles Bolick, Holston Medical Group Pc, Bristol, TN; Rachel Bowers, Palm Research Center Inc., Las Vegas, NV; John Branch, Integrated Research Group, Inc, Riverside, CA; Ken Brantley, Amarillo Medical Specialists, Amarillo, TX; Elizabeth Bretton, Albuquerque Clinical Trials, Inc., Albuquerque, NM; Shannon Brewington, Meridien Research, Brooksville, FL; Andrew Brockmyre, Holston Medical Group Pc, Bristol, TN; Monica Brown, Valley Clinical Trials, Northridge, CA; Wallace F. Burroughs, Athens Medical Group, Athens, TN; Edward Busick Jr, Massachusetts Research, LLC, Waltham, MA; Shana Camp, Protenium Clinical Research, Hurst, TX; Rafael Canadas, Galenos Research, Dallas, TX; Lalaine D. Canete-Kisiel, Clinical Investigation Specialists, Inc., Kenosha, WI; Mary Cao, Diabetes and Thyroid Center of Fort Worth, Fort Worth , TX; Jonna Capuchino, Meridien Research, Brooksville, FL; Crystal Cardwell, Albuquerque Clinical Trials, Inc., Albuquerque, NM; Kristin Carlton, American Health Network of Indiana, LLC, Greenfield, IN; Matthew Cartwright, Clinical Research Advantage/ Family Practice Specialists, Phoenix, AZ; Chet Cartwright, Integrated Research Group, Inc, Riverside, CA; Kathleen Carty, CTI Clinical Research Center, Crestview Hills, KY; Kimberely Casagni, Chase Medical Research LLC, Waterbury, CT; Cindy Castro, Protenium Clinical Research, Hurst, TX; Daniel Chappell, Wade Family Medicine, Bountiful, UT; Louis Chaykin, Meridien Research, Bradenton, FL; Theresa Cheyne, Diabetes and Thyroid Center of Fort Worth, Fort Worth , TX; Christopher Chow, Valley Clinical Trials, Northridge, CA; Mark Christiansen, Diablo Clinical Research, Inc., Walnut Creek, CA; Leonard Chuck, Diablo Clinical Research, Inc., Walnut Creek, CA; Brittany Cole, American Health Network of Indiana, LLC, Avon, IN; Brittany T. Collins, Dominion Medical Associates, Richmond, VA; Celia Colon, ICCT Research International Inc, Chicago, IL; Rosa Contreras, Anaheim Clinical Trials, LLC, Anaheim, CA; Beth Corbin, RN, Albuquerque Clinical Trials, Inc., Albuquerque, NM; Francisco Correra, Cotton O'Neil Clinical Research, Topeka, KS; Becky Cota, Amarillo Medical Specialists, Amarillo, TX; Damian Covington, Dominion Medical Associates, Richmond, VA; Linda Cunningham, Southgate Medical Group, LLP, West Seneca, NY; Nancy Curran, Westside Family Medical Center, P.C., Kalamazoo, MI; Bethani Dandois, Westside Family Medical Center, P.C., Kalamazoo, MI; Thomas Davis III, Integrated Research Group, Inc, Riverside, CA; Rajesh Kumar Davit, Sterling Research Group, Ltd., Cincinnati, OH; Gustavo Day, Galenos Research, Dallas, TX; Raymond De La Rosa, Four Rivers Clinical Research Inc., Paducah, KY; Eric Dedeke, Integris Family Care Northwest, Oklahoma City, OK; Elizabeth Delgado, Anaheim Clinical Trials, LLC, Anaheim, CA; Michael DeMicco, Anaheim Clinical Trials, LLC, Anaheim, CA; Michael Dempsey, Endocrine And Metabolic Consultants, Rockville, MD; Lin Denton, Family Medical Assoc, Levittown, PA; Kristen Lara Dewes, Meridien Research, Tampa, FL; Maureen Didonato, Founders Research Corporation, Philadelphia, PA; Philip Diller, Sterling Research Group, Ltd., Cincinnati, OH; Prasad Dodda, Southern Endocrinology Associates PA, Mesquite, TX; Leonard Dodson, Amarillo Medical Specialists, Amarillo, TX; Amanda Donoho, Holston Medical Group, Kingsport, TN; Michelle Dowell, Meridien Research, Bradenton, FL; Steve Duckor, Anaheim Clinical Trials, LLC, Anaheim, CA; Jeffrey Dugas, Cedar-Crosse Research Center, Chicago, IL; Lois Eaglin, The Center for Sexual Health, Metairie, LA; Dana Edwards, Four Rivers Clinical Research Inc., Paducah, KY; Nikisha N. Edwards, Dominion Medical Associates, Richmond, VA; Elliott Eisenbud, NorCal Endocrinology and Internal Medicine, Roseville, CA; Christina Eizensmits, Protenium Clinical Research, Hurst, TX; Susie Estes, Endocrine Research Solutions, Inc., Roswell, GA; John Gary Evans, East Coast Institute for Research ,LLC, Jacksonville, FL; Neil R. Farris, The Research Group of Lexington, LLC, Lexington, KY; Jack Feliciano, Community Clinical Research, Muncie, IN; Dean Felker, American Health Network of Indiana, LLC, Greenfield, IN; Amanda Fidler, American Health Network, Indianapolis, IN; David Bruce Fittingoff, Infosphere Clinical Studies, Inc., West Hills, CA; Cheri Fletcher, West Broadway Clinic, Bluffs, IA; Evelyn Flores, Valley Clinical Trials, Northridge, CA; Hector Flores, Med-Olam Clinical Reseach LLC, St. Pasadena, TX; Christine Joy A. Florida, Anaheim Clinical Trials, LLC, Anaheim, CA; Chelsea Flowe, Valley Research, Fresno, CA; Steven Folkerth, Providence Health Partners-Center For Clin Research, Dayton, OH; Kenneth Fox, Family Medical Assoc, Levittown, PA; Eulalia Francisco, National Research Institute, Los Angeles, CA; Jenny Francisco, National Research Institute, Los Angeles, CA; Mayra Francisco, National Research Institute, Los Angeles, CA; David M. Francyk, Clinical Research Advantage/ Family Practice Specialists, Phoenix, AZ; Linda Frazee, Cotton O'Neil Clinical Research, Topeka, KS; Juan Pablo Frias, National Research Institute, Los Angeles, CA; Athena Friese, INTEGRIS Family Care Yukon, Yukon, OK; Jennifer Gamble, American Health Network of Indiana, LLC, Avon, IN; Sumana Gangi, Southern Endocrinology Associates PA, Mesquite, TX; Raul Gaona, Briggs Clinical Research, LLC, San Antonio, TX; Nina Garcia, Cedar-Crosse Research Center, Chicago, IL; Noemi Garcia, Manati Center For Clinical Research, Manati, PR; Lauren Gentry, Integrated Research Group, Inc, Riverside, CA; Allan Patterson Goldman, Clinical Research Advantage/ Family Practice Specialists, Phoenix, AZ; Laura Golici, UT Southwestern Medical Center at Dallas, Dallas, TX; Antonieta Gonzalez, Briggs Clinical Research, LLC, San Antonio, TX; Yvonne Goodman, Founders Research Corporation, Philadelphia, PA; Robert Goulet-Ingram, Palm Research Center Inc., Las Vegas, NV; Gary Graf, Cotton O'Neil Clinical Research, Topeka, KS; Andrew Green, Kansas City Internal Medicine, Overland Park, KS; Jean Green-Blair, Hanson Clinical Research Center, Port Charlotte, FL; David Gunther, Southgate Medical Group, LLP, West Seneca, NY; Chi Quyet-Duy Ha, Gateway Research Center, Poway, CA; Thu Minh Ha, Gateway Research Center, Poway, CA; Amina Haggag, Anaheim Clinical Trials, LLC, Anaheim, CA; Jaclyn Hake, Macoupin Research Group, Gillespie, IL; Jammal Hammoud, Elite Research Center, Flint, MI; Wafaa Hammoud, Elite Research Center, Flint, MI; Jaison Hampson, ClinSite LLC, Ann Arbor, MI; Lenita Hanson, Hanson Clinical Research Center, Port Charlotte, FL; Mumtaz Haque, Independent Clinical Researchers @ Wolfson Medical Center, Las Vegas, NV; Amanda Harp, Four Rivers Clinical Research Inc., Paducah, KY; Larissa Harrison, Clinical Research Advantage/ Family Practice Specialists, Phoenix, AZ; Sarah Harvey, Clinical Research Advantage/ Family Practice Specialists, Phoenix, AZ; Annamarie Haught, Clinical Research Advantage/ Family Practice Specialists, Phoenix, AZ; Ashley Helton, Holston Medical Group, Kingsport, TN; Gayle Hennekes, Sterling Research Group, Ltd., Cincinnati, OH; Lauren Hernandez, Briggs Clinical Research, LLC, San Antonio, TX; Carols Herrera, Heights Doctors Clinic, Houston, TX; Judy Hiatt, Westside Family Medical Center, P.C., Kalamazoo, MI; Janiece Hicks, Phillips Medical Services, PLLC, Jackson, MS; Jennifer Hoenig, Clinical Investigation Specialists, Inc., Kenosha, WI; Lisa Hoffman, Southgate Medical Group, LLP, West Seneca, NY; Rebecca Hogan, Providence Health Partners-Center For Clin Research, Dayton, OH; Stanley Hsin-Wei-Hsia, National Research Institute, Los Angeles, CA; Edward Hubach, Providence Health Partners-Center For Clin Research, Dayton, OH; Kimberly Hudson, Integrated Research Group, Inc, Riverside, CA; Leticia Huerta, Med-Olam Clinical Reseach LLC, St.Pasadena, TX; Cynthia Huffman, Meridien Research, Tampa, FL; David Huffman, University Diabetes & Endocrine Consultants, Chattanooga, TN; Jason Hughes, University Diabetes & Endocrine Consultants, Chattanooga, TN; Nicole Hulen, Gateway Research Center, Poway, CA; Sharon Infantino, Elite Research Center, Flint, MI; Giovanni Infusino, ICCT Research International Inc, Chicago, IL; Mohammed Imran Iqbal, Infosphere Clinical Studies, Inc., West Hills, CA; Richard A. Jackson, Dominion Medical Associates, Richmond, VA; Melad Jamal, Valley Clinical Trials, Northridge, CA; Curtis Jantzi, Holston Medical Group, Kingsport, TN; MarDiene Jeffs, Wade Family Medicine, Bountiful, UT; Donni Jester, Endocrine Research Solutions, Inc., Roswell, GA; Preetham Jetty, Community Clinical Research, Muncie, IN; Mandy Jones, University Diabetes & Endocrine Consultants, Chattanooga, TN; Elizabeth Joseph, Clinical Investigation Specialists, Inc., Kenosha, WI; Judy Kallman, Integrated Research Group, Inc, Riverside, CA; Paul Katzenstein, Kansas City Internal Medicine, Overland Park, KS; Sharon Kelly, National Research Institute, Los Angeles, CA; Hala Khoulani, Elite Research Center, Flint, MI; Serena Ann Klugh, Palm Research Center Inc., Las Vegas, NV; Julia Gargan Kozma, ClinSite LLC, Ann Arbor, MI; Kimberly Krogman, IVA Research, Cincinnati, OH; Kevin Kuettel, Anaheim Clinical Trials, LLC, Anaheim, CA; Emile LaBranche, The Center for Sexual Health, Metairie, LA; Jodi Lambert, ClinSite LLC, Ann Arbor, MI; Karen Dellinger Leadbitter, CTI Clinical Research Center, Crestview Hills, KY; Paul Lederer, ICCT Research International Inc, Chicago, IL; Jullia Lee, ClinSite LLC, Ann Arbor, MI; Katie Leonard, Meridien Research, Brooksville, FL; Colleen Leonard, North Shore University Health System, Skokie, IL; Linda Swee Chun Lin, Juno Research, LLC, Katy, TX; Ildiko Lingvay, UT Southwestern Medical Center at Dallas, Dallas, TX; Svetlana Lisovskiy, NorCal Endocrinology and Internal Medicine, Roseville, CA; Svetlana Lisovskiy, NorCal Endocrinology and Internal Medicine, San Ramon, CA; Robert Lockwood, INTEGRIS Family Care Yukon, Yukon, OK; Meyling Lopez, Endocrine And Metabolic Consultants, Rockville, MD; Janet L. Lung, Sterling Research Group, Ltd., Cincinnati, OH; Sean Lynd, IVA Research, Cincinnati, OH; Marlene Ma, Integrated Research Group, Inc, Riverside, CA; James Sample Magee, East Coast Institute for Research ,LLC, Jacksonville, FL; Carol Magerus, Integris Family Care Northwest, Oklahoma City, OK; Carol Magerus, INTEGRIS Family Care Yukon, Yukon, OK; Michael Magnotti, Dr. Michael Magnotti, Teaneck, NJ; Joanne Major, Sterling Research Group, Ltd., Cincinnati, OH; Michelle Mallitz, Meridien Research, Bradenton, FL; Jason Manthei, Macoupin Research Group, Gillespie, IL; Isam Marar, West Broadway Clinic, Bluffs, IA; Amanda Mariotti, Chase Medical Research LLC, Waterbury, CT; Melanie Marshall, Integris Family Care Northwest, Oklahoma City, OK; Christina Marshall, Holston Medical Group Pc, Bristol, TN; Paul A. Martin, Providence Health Partners-Center For Clin Research, Dayton, OH; Ana Martinez, ICCT Research International Inc, Chicago, IL; Kelli Maw, Meridien Research, Brooksville, FL; Joanna Maya, Juno Research, LLC, Katy, TX; Christopher L. Maynard, Athens Medical Group, Athens, TN; Vickie McDaniel, American Health Network of Indiana, LLC, Greenfield, IN; Deidre McMullen, Carl R. Meisner Medical Clinic, PLLC, Sugarland, TX; Amy Megerle, Juno Research, LLC, Katy, TX; Carl Meisner, Carl R. Meisner Medical Clinic, PLLC, Sugarland, TX; Ethelina Menchaca, Heights Doctors Clinic, Houston, TX; Sajeev Menon, Kansas City Internal Medicine, Overland Park, KS; Sarah Van Meter, Four Rivers Clinical Research Inc., Paducah, KY; Peter J. Meyers, ClinSite LLC, Ann Arbor, MI; Debbie Mikesell, Wade Family Medicine, Bountiful, UT; Susan Monaco, Anaheim Clinical Trials, LLC, Anaheim, CA; Catherine Montogomery, Clinical Research Advantage/ Family Practice Specialists, Phoenix, AZ; Stephen Mooney, The Research Group of Lexington, LLC, Lexington, KY; Laurie Moore, CTI Clinical Research Center, Crestview Hills, KY; Emily Morawski, Holston Medical Group, Kingsport, TN; Thomas Moretto, American Health Network, Indianapolis, IN; Denise Morgan, The Research Group of Lexington, LLC, Lexington, KY; David Morin, Holston Medical Group, Kingsport, TN; Robert Morley, The Research Group of Lexington, LLC, Lexington, KY; Julie Mullen, Sterling Research Group, Ltd., Cincinnati, OH; Miguel Munoz, Endocrine Research Solutions, Inc., Roswell, GA; Daniel Murak, Southgate Medical Group, LLP, West Seneca, NY; Samer Nakhle, Palm Research Center Inc., Las Vegas, NV; Samer Nakhle, Palm Research Center Inc., Las Vegas, NV; Jonna Nash, Wade Family Medicine, Bountiful, UT; Stacy Newsome, The Research Group of Lexington, LLC, Lexington, KY; Jennifer Nicholas, The Research Group of Lexington, LLC, Lexington, KY; Gayle Nicholas, Wells Institute For Health Awareness, Kettering, OH; Paul Norwood, Valley Research, Fresno, CA; Gary Noworatzky, Southern Endocrinology Associates PA, Mesquite, TX; Thomas O'Connor, American Health Network of Indiana, LLC, Greenfield, IN; Theresa Ohlson-Elliott, Southgate Medical Group, LLP, West Seneca, NY; Florence Orlandoni, Gateway Research Center, Poway, CA; Ramon Ortiz-Carrasquillo, Manati Center For Clinical Research, Manati, PR; Philip Ostrowski, Southgate Medical Group, LLP, West Seneca, NY; Matthew O'Sullivan, Palm Research Center Inc., Las Vegas, NV; Eileen Palace, The Center for Sexual Health, Metairie, LA; Betsey Palal, Palm Research Center Inc., Las Vegas, NV; Olivia Papcostea, UT Southwestern Medical Center at Dallas, Dallas, TX; Anna Parker, Anaheim Clinical Trials, LLC, Anaheim, CA; Carolyn Parshall, Albuquerque Clinical Trials, Inc., Albuquerque, NM; Hilary Partridge, Wade Family Medicine, Bountiful, UT; Naynesh Patel, Wells Institute For Health Awareness, Kettering, OH; Cathaline Pearson, Independent Clinical Researchers @ Wolfson Medical Center, Las Vegas, NV; Candance Percifull, Albuquerque Clinical Trials, Inc., Albuquerque, NM; Andrea Phillips, Phillips Medical Services, PLLC, Jackson, MS; Alicia Olave Pichon, ICCT Research International Inc, Chicago, IL; Jonathan Platt, Chase Medical Research LLC, Waterbury, CT; Samuel Plucker, Holston Medical Group Pc, Bristol, TN; Madhuri Poduri, UT Southwestern Medical Center at Dallas, Dallas, TX; Laurentiu Pop, UT Southwestern Medical Center at Dallas, Dallas, TX; Hannah Porter, Wade Family Medicine, Bountiful, UT; Miguel Posada, Juno Research, LLC, Katy, TX; Candyce Poteet, Holston Medical Group Pc, Bristol, TN; Talessa Powell, American Health Network of Indiana, LLC, Greenfield, IN; Catherine Pritchard, Cotton O'Neil Clinical Research, Topeka, KS; Diana Rabadi-Marar, West Broadway Clinic, Bluffs, IA; Diane Rabideau, Chase Medical Research LLC, Waterbury, CT; Marina Raikhel, Torrance Clinical Research Institute, Inc., Lomita, CA; Karina Ramirez, Anaheim Clinical Trials, LLC, Anaheim, CA; Lianette Ramos, Juno Research, LLC, Katy, TX; John Chip Reed, Endocrine Research Solutions, Inc., Roswell, GA; Alexandra Reiher, North Shore University Health System, Skokie, IL; Susan Reiling, Founders Research Corporation, Philadelphia, PA; Kyle Rickner, INTEGRIS Family Care Yukon, Yukon, OK; Terry Ridge, American Health Network, Indianapolis, IN; Joseph Risser, Gateway Research Center, Poway, CA; Vanessa Rivera, Integrated Research Group, Inc, Riverside, CA; Sylvia Rivera, Briggs Clinical Research, LLC, San Antonio, TX; Samuel Rivera-Flores, Heights Doctors Clinic, Houston, TX; Helena Rodbard, Endocrine And Metabolic Consultants, Rockville, MD; Alfredo Rodes, Southgate Medical Group, LLP, West Seneca, NY; Karina Rodriguez, Cedar-Crosse Research Center, Chicago, IL; Brenda Rodriguez, Southern Endocrinology Associates PA, Mesquite, TX; Rebecca Rosenwasser, East Coast Institute for Research, LLC, Jacksonville, FL Tammy Lynn Ross, East Coast Institute for Research, LLC, Fleming Island, FL; Shasta Rowe, Endocrine Research Solutions, Inc., Roswell, GA; Gary Ruoff, Westside Family Medical Center, P.C., Kalamazoo, MI; Holly D. Russell, Meridien Research, Brooksville, FL; Gordon Sack, Med-Olam Clinical Reseach LLC, St. Pasadena, TX; David Sacks, Anaheim Clinical Trials, LLC, Anaheim, CA; Rafik Saleh, Integrated Research Group, Inc, Riverside, CA; Jinna Santiago, Manati Center For Clinical Research, Manati, PR; Daisy Santiago, Manati Center For Clinical Research, Manati, PR; Tabby Sapp, Endocrine Research Solutions, Inc., Roswell, GA; Joanna Schiferl, Cedar-Crosse Research Center, Chicago, IL; Mary Schmoll, American Health Network of Indiana, LLC, Avon, IN; Laurie Ann Schweppe, Meridien Research, Bradenton, FL; Laurie Ann Schweppe, Meridien Research, Brooksville, FL; Samantha Secord, Westside Family Medical Center, P.C., Kalamazoo, MI; Scott Alan Segel, East Coast Institute for Research ,LLC, Jacksonville, FL; Roberto Segura, ICCT Research International Inc, Chicago, IL; Marigene Salazar Sharma, Albuquerque Clinical Trials, Inc., Albuquerque, NM; Charles Sharpe, Athens Medical Group, Athens, TN; Kathyrn Sheridan, Clinical Research Advantage/ Family Practice Specialists, Phoenix, AZ; Rita Sheridan, Hanson Clinical Research Center, Port Charlotte, FL; Kristin Y. Shimazaki, Torrance Clinical Research Institute, Inc., Lomita, CA; Yshay Shlesinger, NorCal Endocrinology and Internal Medicine, Roseville, CA; Deborah Shockley, Providence Health Partners-Center For Clin Research, Dayton, OH; Ricardo A. Silva, East Coast Institute for Research ,LLC, Jacksonville, FL; icardo Silva, East Coast Institute for Research, LLC, Fleming Island , FL; Raj Sindher, Integrated Research Group, Inc, Riverside, CA; Edith Sisneros, Infosphere Clinical Studies, Inc., West Hills, CA; Brian Snyder, Southgate Medical Group, LLP, West Seneca, NY; Dana Soetaert, Cotton O'Neil Clinical Research, Topeka, KS; Jose Solorio, Galenos Research, Dallas, TX; Amy Soto, Palm Research Center Inc., Las Vegas, NV; Joseph Soufer, Chase Medical Research LLC, Waterbury, CT; Tyler John Southwell, Clinical Research Advantage/ Family Practice Specialists, Phoenix, AZ; Helen Sparks, Hanson Clinical Research Center, Port Charlotte, FL; Helen Lee Stacy, Diablo Clinical Research, Inc., Walnut Creek, CA; Ronald Stegemoller, American Health Network of Indiana, LLC, Avon, IN; Deborah Stitt, Holston Medical Group Pc, Bristol, TN; Kassi Stockert, INTEGRIS Family Care Yukon, Yukon, OK; Dennis Stone, Family Medical Assoc, Levittown, PA; Dan Alexandru Streja, Infosphere Clinical Studies, Inc., West Hills, CA; David Streja, Infosphere Clinical Studies, Inc., West Hills, CA; Robert Strzinek, Protenium Clinical Research, Hurst, TX; Danny Sugimoto, Cedar-Crosse Research Center, Chicago, IL; Senan Sultan, East Coast Institute for Research ,LLC, Jacksonville, FL; Senan Sultan, East Coast Institute for Research, LLC, Fleming Island , FL; Kristy Sunada, Diablo Clinical Research, Inc., Walnut Creek, CA; Glen Sussman, ICCT Research International Inc, Chicago, IL; David Sutton, East Coast Institute for Research ,LLC, Jacksonville, FL; Jennifer Swan, Macoupin Research Group, Gillespie, IL; Claude Sy, Southgate Medical Group, LLP, West Seneca, NY; Istavan Szilagyi, East Coast Institute for Research, LLC, Fleming Island , FL; Anjanette Tan, Diabetes and Thyroid Center of Fort Worth, Fort Worth , TX; Jessica Tapia, Endocrine Research Solutions, Inc., Roswell, GA; Peggy Taylor, Holston Medical Group, Kingsport, TN; Elizabeth Taylor, Massachusetts Research, LLC, Waltham, MA; Jessica Terry, Protenium Clinical Research, Hurst, TX; Donna Thomas, IVA Research, Cincinnati, OH; Karen Thomas, American Health Network, Indianapolis, IN; Letitia Thompson-Hargrave, Wells Institute For Health Awareness, Kettering, OH; Florence Toe, Carl R. Meisner Medical Clinic, PLLC, Sugarland, TX; Sara Toler, Protenium Clinical Research, Hurst, TX; Adam Toomey, Westside Family Medical Center, P.C., Kalamazoo, MI; Carole Torello, Chase Medical Research LLC, Waterbury, CT; Hugo Toro, Juno Research, LLC, Katy, TX; Julie Toven, Valley Clinical Trials, Northridge, CA; Paige Trausch, West Broadway Clinic, Bluffs, IA; Claudia Tufts, CTI Clinical Research Center, Crestview Hills, KY; Christina Valdez, Heights Doctors Clinic, Houston, TX; Anna Vanderheiden, UT Southwestern Medical Center at Dallas, Dallas, TX; Raghupathy Varavenkataraman, Southgate Medical Group, LLP, West Seneca, NY; Laura Villegas, Palm Research Center Inc., Las Vegas, NV; Ralph David Wade, Wade Family Medicine, Bountiful, UT; Khurram Wadud, East Coast Institute for Research, LLC, Fleming Island , FL; Robert Joseph Wagner, CTI Clinical Research Center, Crestview Hills, KY; Sandra Waite, Wade Family Medicine, Bountiful, UT; Jesse Wallace, Four Rivers Clinical Research Inc., Paducah, KY; Debra Ward, Kansas City Internal Medicine, Overland Park, KS; Sandy Warr, Wade Family Medicine, Bountiful, UT; Bruce Weber, Macoupin Research Group, Gillespie, IL; Richard Weinstein, Diablo Clinical Research, Inc., Walnut Creek, CA; Sarah Weiss, Meridien Research, Bradenton, FL; Matthew Wenker, Sterling Research Group, Ltd., Cincinnati, OH; Howard Wenocur, Founders Research Corporation, Philadelphia, PA; Bethany Wexler, Diablo Clinical Research, Inc., Walnut Creek, CA; Rick Whiles, Holston Medical Group Pc, Bristol, TN; Connie White, Wade Family Medicine, Bountiful, UT; Judith White, Holston Medical Group, Kingsport, TN; Mark Wiesen, Dr. Michael Magnotti, Teaneck, NJ; Raechelle Wilkins, Westside Family Medical Center, P.C., Kalamazoo, MI; Jody Williams, Wells Institute For Health Awareness, Kettering, OH; Rebecca Williams, Holston Medical Group Pc, Bristol, TN; Robyn Wilson, Heights Doctors Clinic, Houston, TX; Peter Winkle, Anaheim Clinical Trials, LLC, Anaheim, CA; Eric Wolfson, Independent Clinical Researchers @ Wolfson Medical Center, Las Vegas, NV; Nancy Jean Wood, Clinical Research Advantage/ Family Practice Specialists, Phoenix, AZ; Cynthia Wright, Sterling Research Group, Ltd., Cincinnati, OH; Alan Wynne, Cotton O'Neil Clinical Research, Topeka, KS; Mark A. Wyse, Clinical Research Advantage/ Family Practice Specialists, Phoenix, AZ; Gary D. Yeoman, Founders Research Corporation, Philadelphia, PA; Janet Yoo, North Shore University Health System, Skokie, IL; Daniel Yousef, Clinical Investigation Specialists, Inc., Kenosha, WI; Encarna Zamanian, Integrated Research Group, Inc, Riverside, CA.

## Supplementary Material 1: Rescue criteria and medication

- Subjects with fasting self-measured plasma glucose values exceeding predefined limits repeated the measurement on two consecutive days; if the mean of these three measurements, or any fasting plasma glucose measured by the central laboratory, exceeded predefined limits without an apparent cause, the subject was invited for an unscheduled visit as soon as possible. If a confirmatory fasting plasma glucose also exceeded predefined limits, the subject was offered rescue medication as an add-on to their randomized treatment
- Rescue medication involved intensification of existing background medication and/or initiation of new medication with basal insulin dose increase as the preferred first choice

## Supplementary Material 2: Events adjudicated by the external Event Adjudication Committee

The following adverse events were judged in this trial by an external Event Adjudication Committee:

- Fatal events (if not covered by another medical event of special interest)
- Acute coronary syndrome
- Cerebrovascular event (stroke or transient ischemic attack)
- Coronary revascularization procedure
- Heart failure requiring hospital admission
- Neoplasm (excluding thyroid neoplasm)
- Thyroid disease (including thyroid neoplasm or resulting in thyroidectomy)
- Pancreatitis or clinical symptoms leading to suspicion of pancreatitis
  - Pancreatitis was confirmed by fulfilling, as a minimum, 2 out of the following 3 criteria: i) severe acute abdominal pain; ii) amylase and/or lipase >3× upper limit of normal; iii) characteristic findings on relevant imaging e.g. computerized axial tomography (CT)/MRI/ultrasound)

## Supplementary Material 3: Details of statistical analysis

The trial was designed to establish superiority jointly for both doses of semaglutide versus pooled placebo (hereafter referred to as placebo) for the change in HbA_1c_ and body weight at Week 30 with a one-sided alpha of 2.5%, assuming treatment differences versus placebo of 0.45% and 2.25 kg, respectively for each semaglutide dose level and SDs of 1.1% and
4.0 kg, respectively. The overall power to simultaneously demonstrate superiority for the two dose levels of semaglutide versus placebo in HbA_1c_ and body weight was 82%. In order to preserve the overall type I error, the conclusion of superiority with treatment of each semaglutide dose versus placebo after 30 weeks was evaluated hierarchically. The superiority of semaglutide change in HbA_1c_ needed to be established before superiority in change in body weight could be evaluated.

HbA_1c_, body weight and other continuous endpoints assessed over time were analyzed using a mixed model for repeated measurements with treatment, country and stratification variables (HbA_1c_ level at screening [up to 8.0% or less than 8.0%] crossed with use of metformin [yes or no]; 2 by 2 levels) as fixed factors and baseline value as covariate, all nested within visit. An unstructured covariance matrix was assumed for measurements within the same subject. Responder outcomes were analyzed using logistic regression.

The robustness of the analyses of HbA_1c_ and body weight was assessed by handling missing data in various ways, including a pattern mixture model where missing data points were imputed as if the subject was receiving placebo. Sensitivity analyses also included a mixed model for repeated measurements analysis on the full analysis set using all data regardless of whether it was obtained while the subjects had discontinued the trial product and/or whether the subject had been administered rescue medication (Supplementary Material 4).

## Supplementary Material 4: The sensitivity of the primary and confirmatory secondary endpoint was assessed in four separate sensitivity analyses

- Complete case analysis: Analysis of the mixed model for repeated measurements based on data only from subjects who completed the trial and did not receive rescue medication
- In-trial analysis (mixed model for repeated measurements): Analysis of the mixed model for repeated measurements based on all data recorded after randomization, regardless of adherence to randomized treatment or initiation of rescue therapy
- Analysis of covariance (last observation carried forward): Analysis of covariance using imputation of missing values according to the last-observation-carried-forward method based on the subjects who were on treatment and did not take rescue medications
- Pattern mixture model: Analysis of the pattern mixture model, mimicking an intention-to-treat scenario, where withdrawn subjects are assumed to be switched to the control treatment after withdrawal

# Supplementary figures

## Figure S1. Trial design


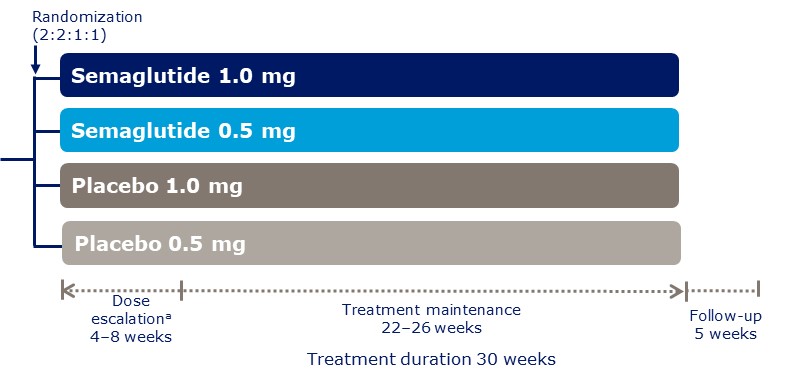


^a^Dose escalation from starting dose of 0.25 mg, dose doubled each step until trial dose achieved

## Figure S2: Subject disposition (CONSORT)


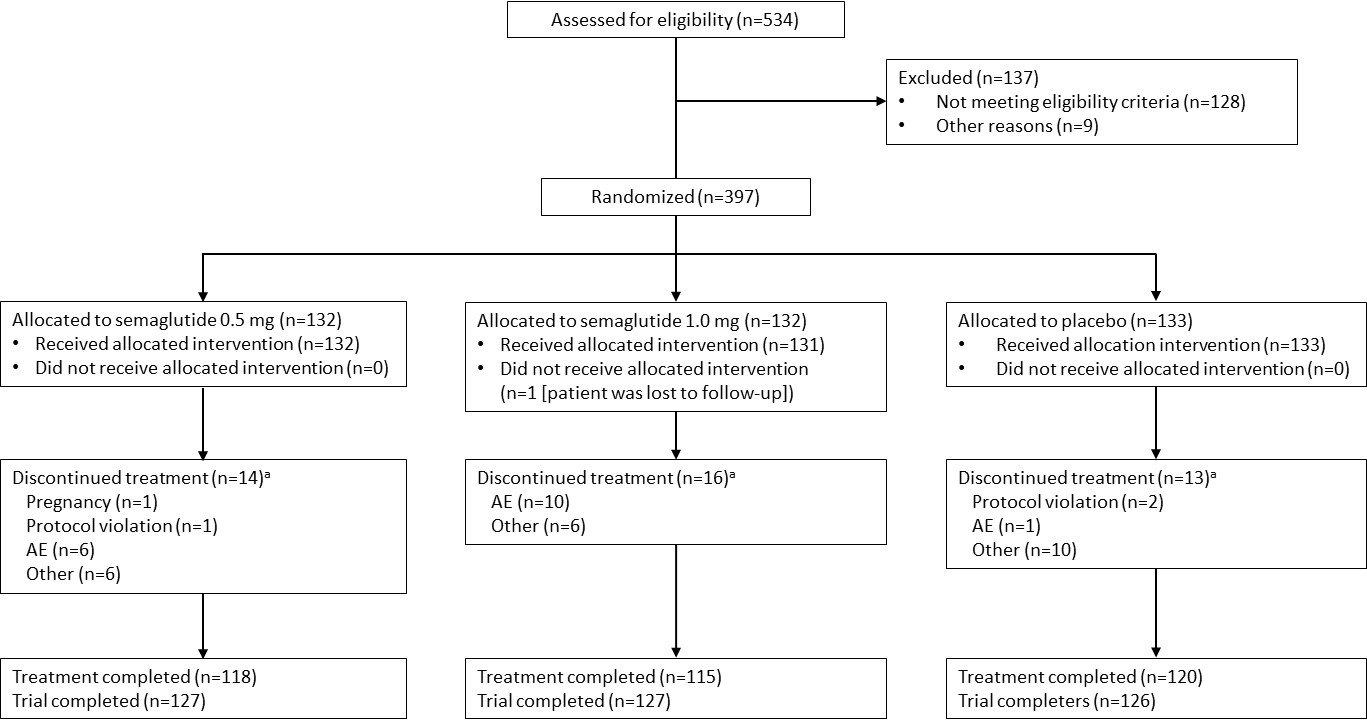


^a^Reflects primary reason for treatment discontinuation, as judged by the investigator.

The top four reasons for not meeting the eligibility criteria were: HbA_1c_ levels outside the required range; not meeting diagnosis and treatment requirements (subjects diagnosed with type 2 diabetes and on stable diabetes treatment (± 20% change in total daily dose) with basal insulin [minimum of 0.25 IU kg^–1^ day^–1^ and/or 20 IU/day of: insulin glargine, insulin detemir, insulin degludec and/or neutral protamine Hagedorn insulin] alone or in combination with metformin [minimum of 1500 mg/day or maximal tolerable dose] for 90 days prior to screening); meeting the exclusion criteria regarding severe renal impairment (estimated glomerular filtration rate <30 ml min^–1^ 1.73 m^–2^ per Modification of Diet in Renal Disease formula; treatment with any glucose-lowering agents other than stated in the inclusion criteria in a period of 90 days before screening [exception: short-term treatment (≤7 days in total) with bolus insulin in connection with intercurrent illness]; or known proliferative retinopathy or maculopathy requiring acute treatment according to the opinion of the investigator. AE, adverse event; HbA_1c_, glycated hemoglobin.

## Figure S3. Sensitivity analyses

(A) Change in HbA_1c_

^
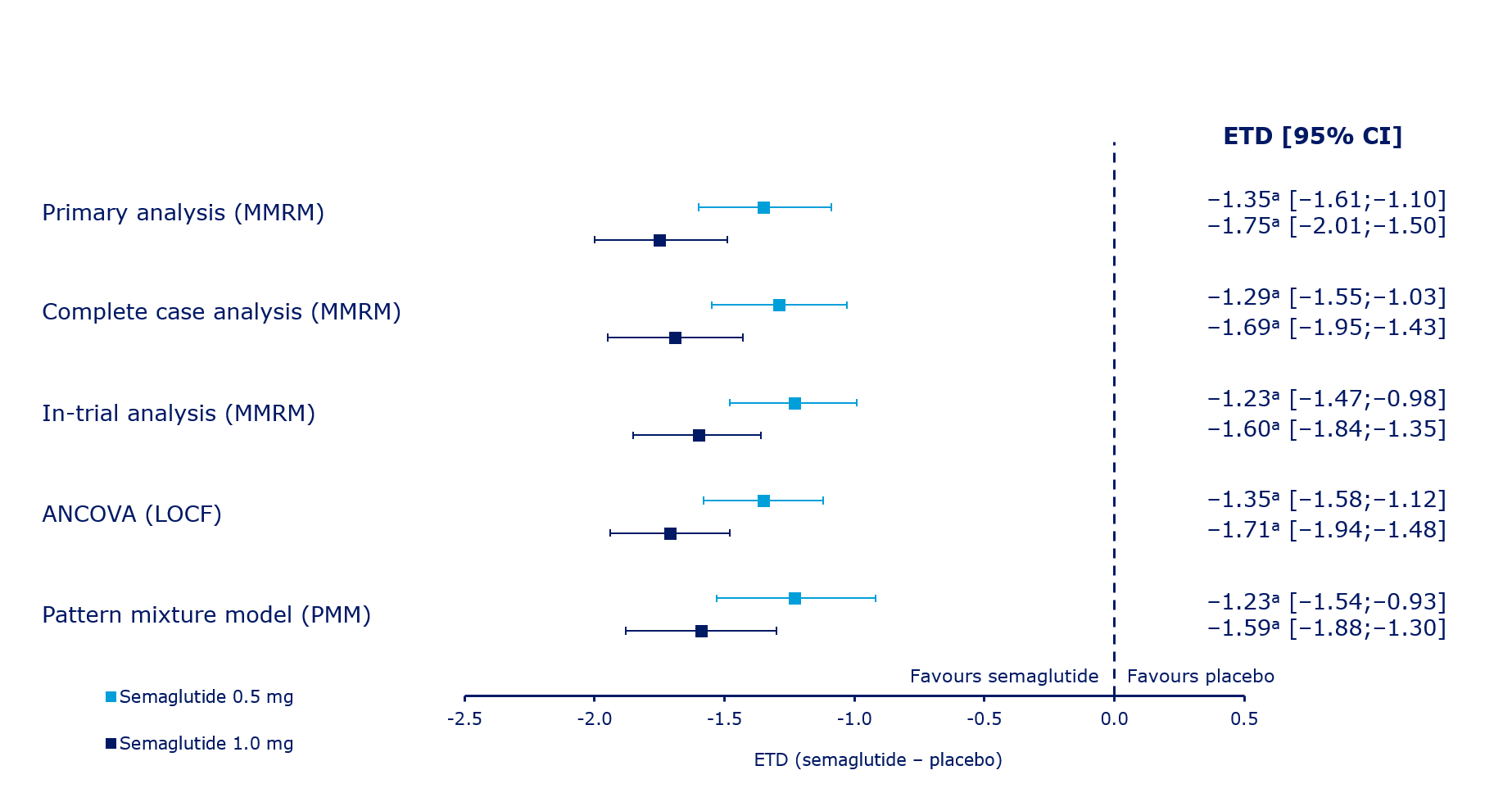
^

^a^Indicates significance (p<0.0001). ETD, estimated treatment difference; HbA_1c_, glycated hemoglobin; LOCF, last observation carried forward; MMRM, mixed model for repeated measurements analysis.

(B) Change in body weight

^
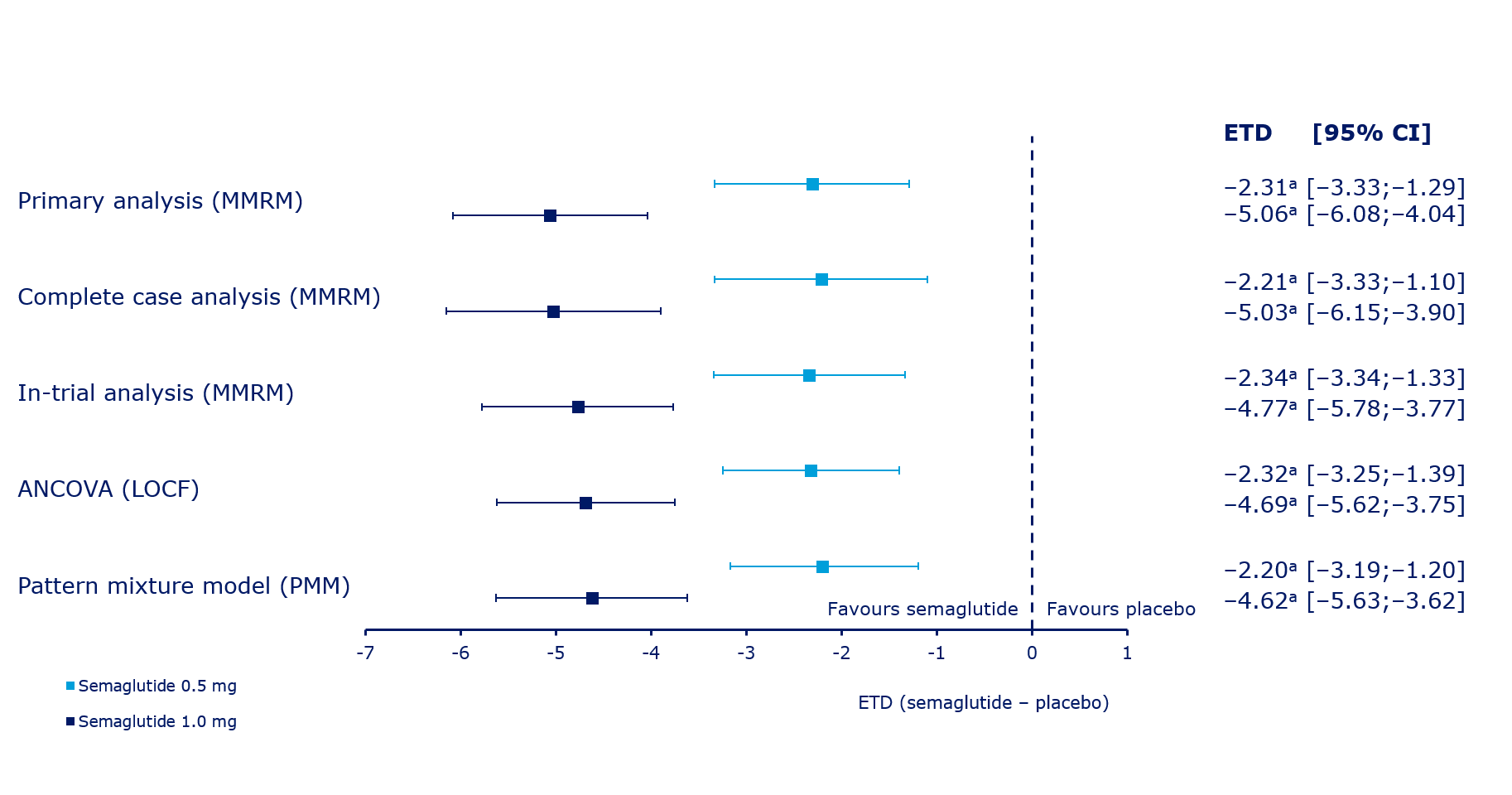
^

^a^Indicates significance (p≤0.0001). ETD, estimated treatment difference; HbA_1c_, glycated hemoglobin; LOCF, last observation carried forward; MMRM, mixed model for repeated measurements analysis.

## Figure S4. Subjects achieving (A) a HbA_1c_ target of ≤6.5% (American Association of Clinical Endocrinologists target) and (B) a body weight loss target of ≥10% at Week 30

(A)


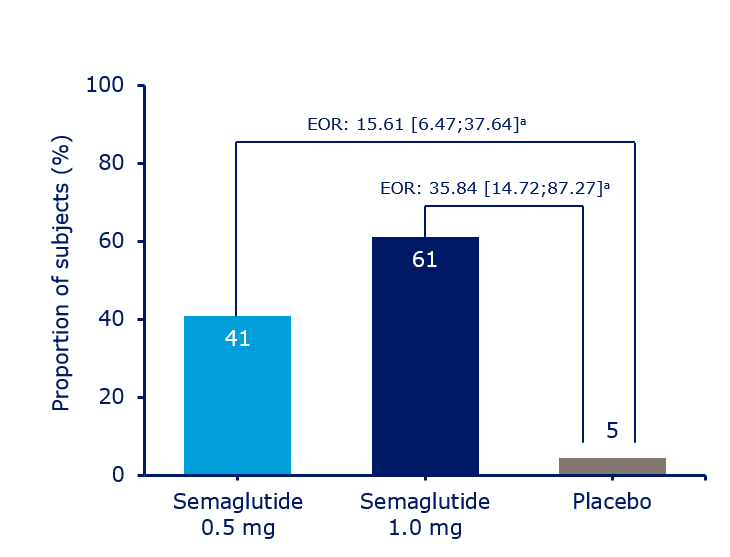


(B)


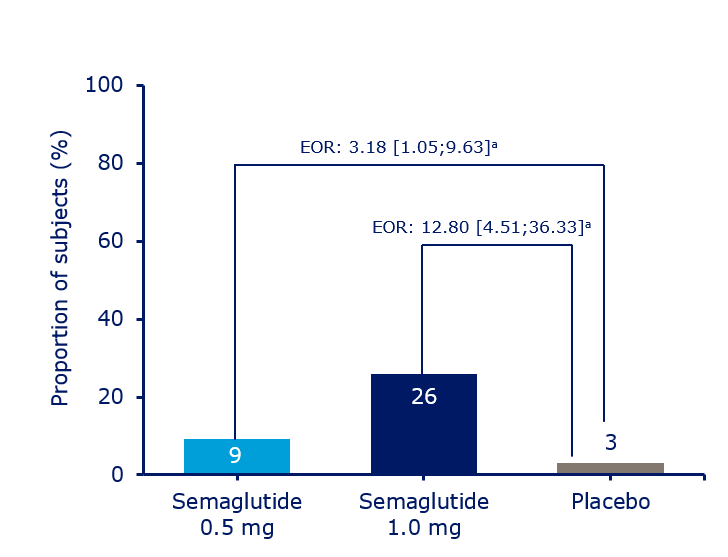


^a^Indicates significance (p<0.05). Values are observed proportions using ‘on-treatment without rescue medication’ data from subjects in the full analysis set. Missing HbA_1c_ and body weight data are imputed from a mixed model for repeated measurements analysis and subsequently classified. Values in square brackets indicate 95% CIs. BMI, body mass index; HbA_1c_, glycated hemoglobin; EOR, estimated odds ratio.

## Figure S5. Insulin dose: observed ratio to baseline by week for subjects with HbA_1c_ (A) ≤8.0% or (B) >8% at randomization

(A)


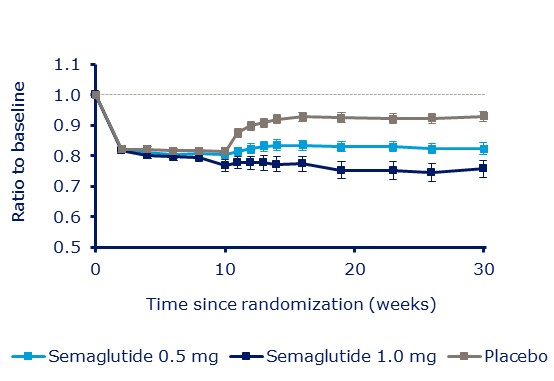


(B)


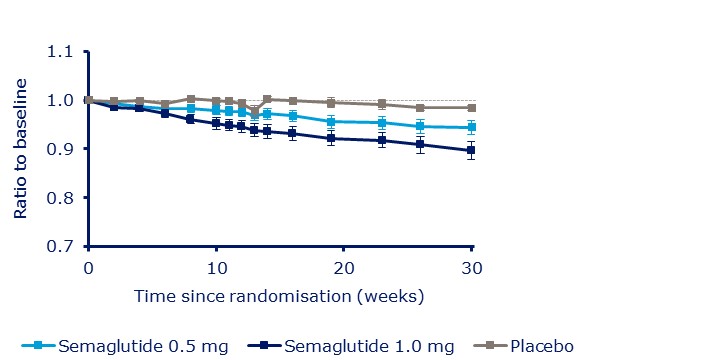


Values are observed means (± SEs) from a mixed model for repeated measurements analysis using ‘on-treatment without rescue medication’ data from subjects in the full analysis set. Dashed line indicates the overall mean value at baseline. HbA_1c_, glycated hemoglobin.

## Figure S6. Subject-reported outcomes: change in Diabetes Treatment Satisfaction Questionnaire(s) compared with placebo for semaglutide 0.5 mg and semaglutide 1.0 mg


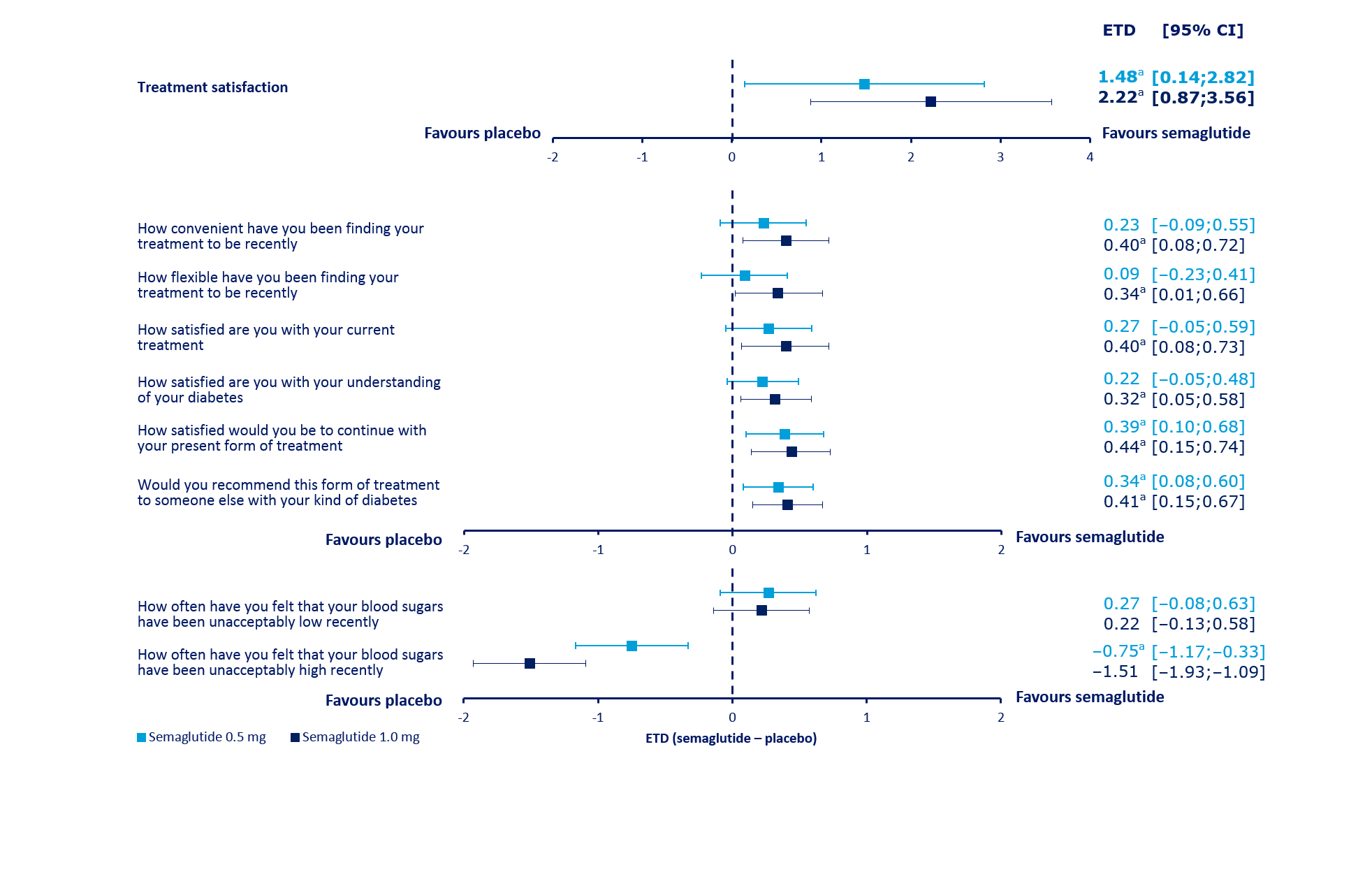


^a^Indicates significance (p≤0.04). Values are ETDs with 95% CIs from a mixed model for repeated measurements analysis using ‘on-treatment without rescue medication’ data from subjects in the full analysis set. The 8 DTSQ items are scored from 0 to 6. Treatment Satisfaction is the sum of 6 of the 8 DTSQ items, and ranges from 0 to 36.
DTSQ, Diabetes Treatment Satisfaction Questionnaire; ETD, estimated treatment difference.

## Figure S7. Prevalence of (A) nausea and (B) vomiting events

(A) Prevalence of nausea events


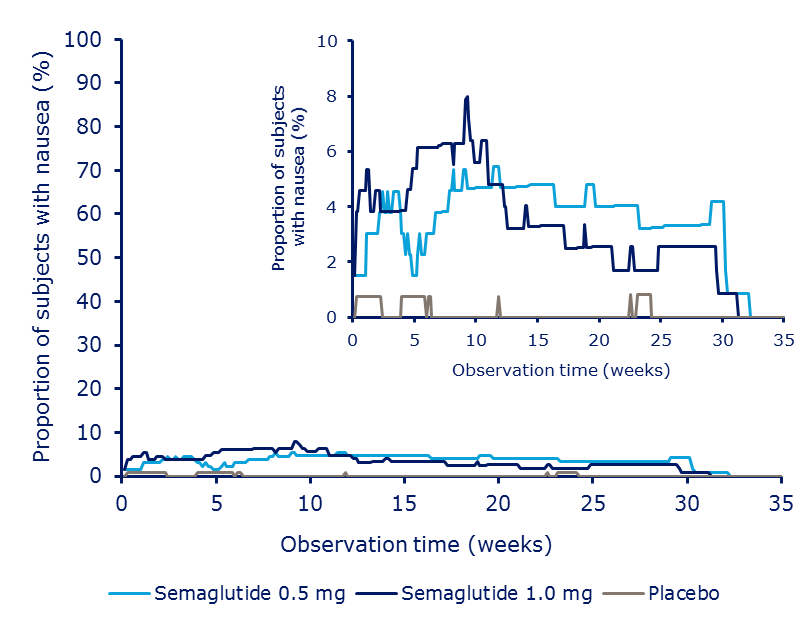


(B) Prevalence of vomiting events


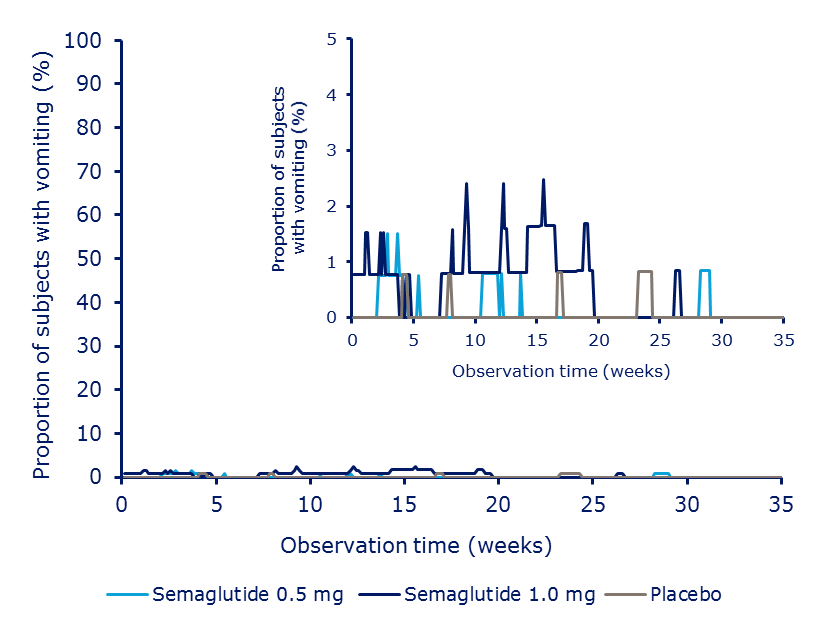


‘On-treatment’ summary of adverse events. Events are shown until the scheduled follow-up visit (5 weeks after last dose).

## Figure S8. Time to onset of first severe or blood glucose-confirmed symptomatic hypoglycemic event


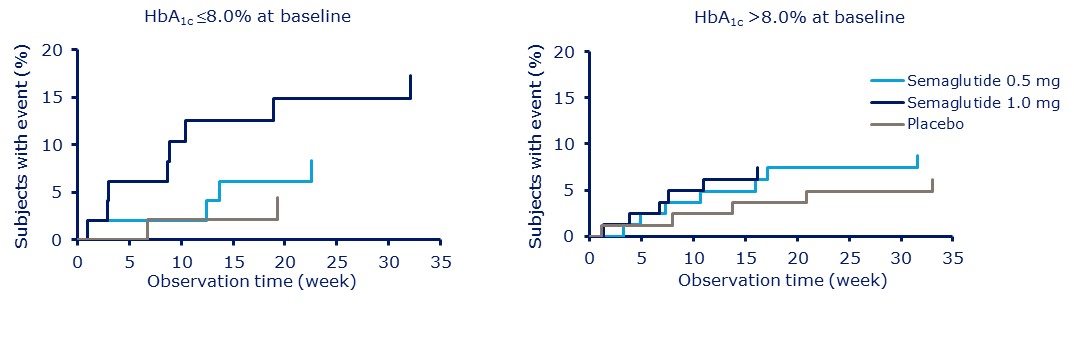


HbA_1c_, glycated hemoglobin.

## Figure S9. Lipase (A) and amylase (B) estimated mean levels by week and ratio to baseline at Week 30

(A)


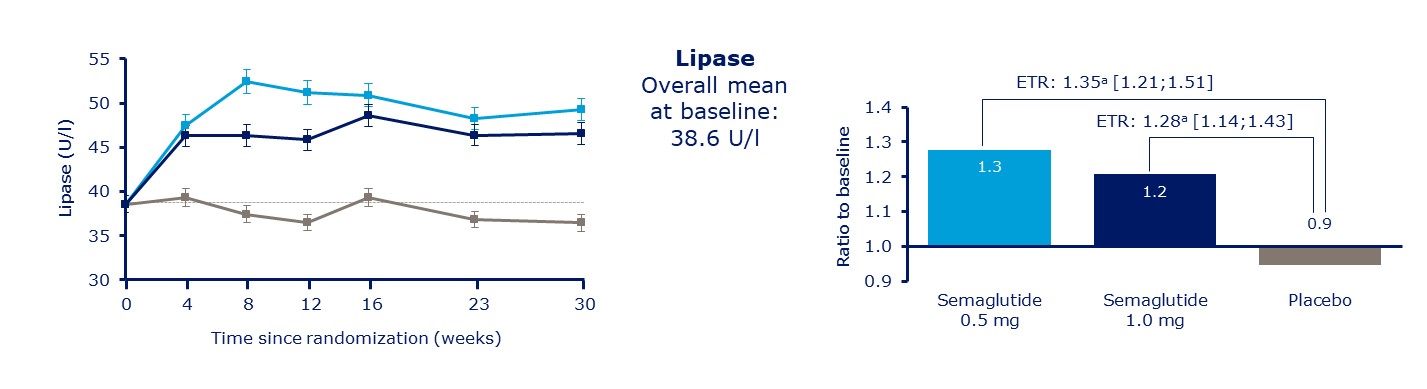


(B)


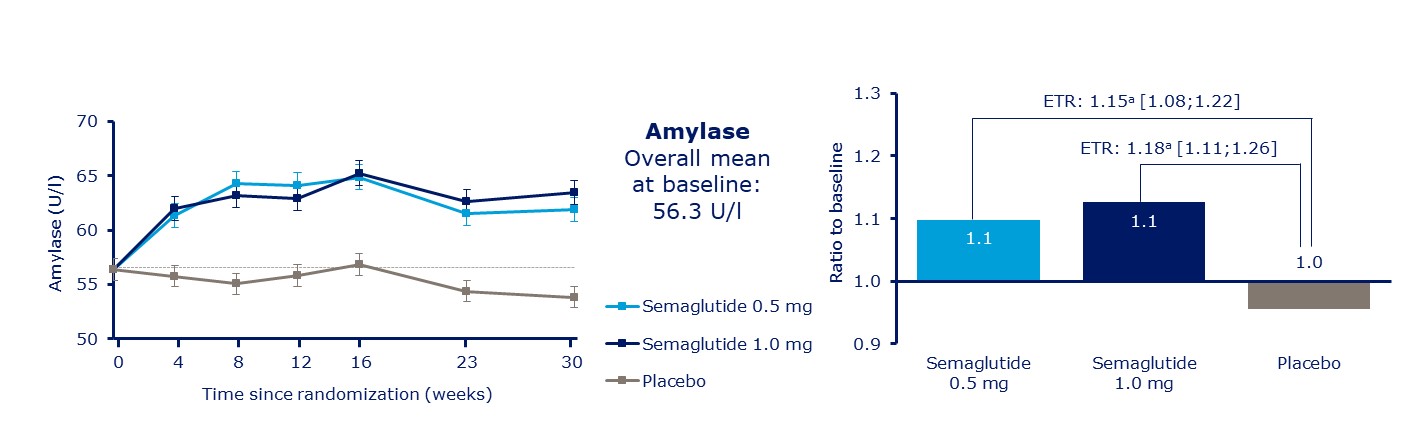


^a^Indicates significance (p<0.0001). Values are estimated means (± SEs) from a mixed model for repeated measurements analysis using ‘on-treatment’ data from subjects in the safety analysis set. Dashed line indicates the overall mean value at baseline. Values in square brackets indicate 95% CIs. ETR, estimated treatment ratio.

# Supplementary tables

## Table S1. Inclusion and exclusion criteria

| Inclusion criteria   - Informed consent obtained before any trial-related activities. Trial-related activities are any procedures that are carried out as part of the trial, including activities to determine suitability for the trial - Male or female, age ≥18 years at the time of signing inform consent. For Japan: male or female, age ≥20 years at the time of signing informed consent - Subjects diagnosed with type 2 diabetes and on stable diabetes treatment (up to 20% increase or decrease in total daily dose) with basal insulin (minimum of  0.25 IU kg^–1^ day^–1^ and/or 20 IU/day of: insulin glargine, insulin detemir, insulin degludec and/or Neutral Protamine Hagedorn insulin) alone or in combination with metformin (minimum of 1500 mg/day or maximal tolerable dose) for 90 days prior  to screening - HbA_1c_ 53.0–85.8 mmol/mol (7.0–10.0%) both inclusive   Exclusion criteria   - Known or suspected hypersensitivity to trial product(s) or related products - Previous participation in this trial. Participation is defined as signed informed consent - Female who is pregnant, breastfeeding or intends to become pregnant or is of child-bearing potential and not using an adequate contraceptive method throughout the trial including the 5-week follow-up period (adequate contraceptive measures as required by local regulation or practice). Germany: Only highly effective methods of birth control are accepted (i.e. one that results in less than 1% per year failure rate when used consistently and correctly such as implants, injectables, combined oral contraceptives, some intrauterine devices), or sexual abstinence or vasectomized partner. Japan: Adequate contraceptive measures are abstinence, diaphragm, condom (by the partner), intrauterine device, sponge, spermicide or oral contraceptive - Receipt of any investigational medicinal product within 90 days before screening - Any chronic disorder or severe disease which, in the opinion of the investigator, might jeopardize subject’s safety or compliance with the protocol - Treatment with any glucose-lowering agents other than stated in the inclusion criteria in a period of 90 days before screening. An exception is short-term treatment (7 days or fewer in total) with bolus insulin in connection with intercurrent illness - Experience of more than 3 episodes of severe hypoglycemia within 6 months prior to screening, and/or hypoglycemia unawareness - History of pancreatitis (acute or chronic) - Screening calcitonin value 50 ng/l (50 pg/ml) or greater - Personal or family history of medullary thyroid carcinoma or multiple endocrine neoplasia type 2 - Severe renal impairment defined as an estimated glomerular filtration rate below  30 ml min^–1^ 1.73 m^–2^ per Modification of Diet in Renal Disease formula (4 variable version) - Acute coronary or cerebrovascular event within 90 days before randomization - Heart failure, New York Heart Association Class IV - Known proliferative retinopathy or maculopathy requiring acute treatment according to the opinion of the investigator - Diagnosis of malignant neoplasm in the previous 5 years (except basal cell skin cancer or squamous cell skin cancer) - Mental inability, unwillingness, or language barrier precluding adequate understanding of or compliance with study procedures |
| --- |

## Table S2. Insulin titration during the study

| **Decrease in insulin** | | | | |
| --- | --- | --- | --- | --- |
| **Lowest fasting plasma glucose** | | | | **Adjustment of basal insulin**  **(total daily dose) IU** |
| **mmol/l** | | **mg/dl** | |  |
| <3.1 | | <56 | | –4 (for doses >45 IU, suggest dose reduction of 10%) |
| ≥3.1 – <4.0 | | ≥56 – <71 | | –2 (for doses >45 IU, suggest dose reduction of 5%) |
| Increase of insulin | | | | |
| **Lowest fasting plasma glucose** | | | **Adjustment of basal insulin**  **(total daily dose) IU** | |
| **mmol/l** | **mg/dl** | |  |  |
| ≥4.0 – <5.5 | ≥71 – <100 | | No adjustment | |
| ≥5.5 – <6.7 | ≥100 – <120 | | +0–2 (at the discretion of the investigator) | |
| ≥6.7 – <7.8 | ≥120 – <140 | | +2 | |
| ≥7.8 – <10.0 | ≥140 – <180 | | +4 | |
| ≥10.0 | ≥180 | | +6–8 (at the discretion of the investigator) | |

Note, for subjects with an HbA_1c_ <8%: insulin could be up-titrated from Weeks 10 to 16, based on the lowest of three consecutive fasting SMBG values. For all subjects: from
Weeks 0 to 12, the insulin dose could be down-titrated according to the above table based on the lowest of three consecutive fasting SMBG readings. For insulin glargine, insulin detemir, and insulin degludec the unit is U; for NPH insulin the unit is IU. For the purpose of analyzing insulin dose, U and IU are not differentiated in this document. HbA_1c_, glycated hemoglobin; IU, international units; NPH, neutral protamine Hagedorn; SMBG, self-measured blood glucose.

## Table S3. Change from baseline to Week 30 in primary and secondary efficacy outcomes by treatment group

|  | **Overall baseline** | **Semaglutide 0.5 mg**  **n=132** | | | **Semaglutide 1.0 mg**  **n=131** | | | **Placebo (n=133)** |
| --- | --- | --- | --- | --- | --- | --- | --- | --- |
|  | Mean  [SD] | Change from baseline [SE] | ETD  [95% CI] | p | Change from baseline [SE] | ETD  [95% CI] | p | Change from baseline [SE] |
| **Glycemic endpoints** | | | | | | | | |
| **HbA_1c,_ mmol/mol** | 67.9  [9.2] | –15.95 [11.76] | –14.79  [–17.54; –12.03] | <0.0001 | –20.41  [9.95] | –19.18  [–21.95; –16.42] | <0.0001 | –2.09  [11.74] |
| **HbA_1c,_ %** | 8.4  [0.84] | –1.4  [0.09] | –1.35  [–1.61; –1.10] | <0.0001 | –1.8  [0.09] | –1.75  [–2.01; –1.50] | <0.0001 | –0.1  [0.09] |
| **Fasting plasma glucose, mmol/l** | 8.6  [2.98] | –1.6  [0.21] | –1.14  [–1.75; –0.54] | 0.0002 | –2.4  [0.21] | –1.88  [–2.48; –1.28] | <0.0001 | –0.5  [0.22] |
| **Fasting plasma glucose, mg/dl** | 155.9  [53.68] | –29.1  [3.74] | –20.62  [–31.45; –9.80] | 0.0002 | –42.4  [3.76] | –33.87  –44.69; –23.06] | <0.0001 | –8.5  [4.02] |
| **7-point self-measured plasma glucose, mmol/l** | | | | | | | | |
| Increment | 3.1  [2.39] | –0.8  [0.15] | –0.66  [–1.10; –0.23] | 0.0030 | –1.2  [0.15] | –1.01  [–1.44; –0.58] | <0.0001 | –0.2  [0.16] |
| Mean | 11.2  [2.77] | –2.5  [0.20] | –1.76  [–2.32; 1.19] | <0.0001 | –3.0  [0.20] | –2.28  [–2.84; –1.72] | <0.0001 | –0.8  [0.21] |
| **7-point self-measured plasma glucose, mg/dl** | | | | | | | | |
| Increment | 55.5  [43.12] | –15.0  [2.72] | –11.92  [–19.75; –4.08] | 0.0030 | –21.3  [2.71] | –18.16  [–25.93; –10.38] | <0.0001 | –3.1  [2.88] |
| Mean | 201.5  [49.85] | –45.4  [3.55] | –31.65  [–41.81; –21.48] | <0.0001 | –54.8  [3.55] | –41.04  [–51.16; –30.91] | <0.0001 | –13.8  [3.74] |
| **Body weight endpoints** | | | | | | | | |
| **Weight, kg** | 91.7  [20.97] | –3.7  [0.36] | –2.31  [–3.33; –1.29] | <0.0001 | –6.4  [0.36] | –5.06  [–6.08; –4.04] | <0.0001 | –1.4  [0.37] |
| **BMI, kg/m^2^** | 32.2  [6.16] | –1.3  [0.12] | –0.84  [–1.20; –0.49] | <0.0001 | –2.3  [0.13] | –1.82  [–2.18; –1.47] | <0.0001 | –0.5  [0.13] |
| **Waist circumference, cm** | 108.3  [15.29] | –3.5  [0.48] | 1.46  [–2.83; –0.09] | 0.0365 | –6.0  [0.49] | –4.05  [–5.42; –2.67] | <0.0001 | –2.0  [0.50] |
| **BP and pulse rate** | | | | | | | | |
| **BP, mmHg** |  |  |  |  |  |  |  |  |
| Systolic | 134.8  [15.98] | –4.3  [1.26] | –3.31  [–6.92; 0.31] | 0.0728 | –7.3  [1.27] | –6.29  [–9.91; –2.66] | 0.0007 | –1.0  [1.34] |
| Diastolic | 79.0  [9.79] | –1.8  [0.73] | 0.33  [–1.80; 2.45] | 0.7606 | –1.5  [0.74] | 0.66  [–1.47; 2.80] | 0.5412 | –2.2  [0.79] |
| Pulse rate (bpm)^a^ | 73.5  [10.66] | 0.8  [0.81] | 1.63  [–0.62; 3.88] | 0.1556 | 4.0  [0.82] | 4.74  [2.48; 7.01] | <0.0001 | –0.8  [0.81] |

^a^Safety endpoint. BMI, body mass index; BP, blood pressure; bpm, beats per minute; ETD, estimated treatment difference versus placebo; HbA_1c_, glycated hemoglobin.

## Table S4. Change from baseline to Week 30 in other secondary efficacy outcomes by treatment group

|  | **Overall baseline** | **Semaglutide 0.5 mg** | | | **Semaglutide 1.0 mg** | | | **Placebo** |
| --- | --- | --- | --- | --- | --- | --- | --- | --- |
|  | Geometric mean (CV) | Ratio to baseline [SE] | ETR  [95% CI] | p | Ratio to baseline [SE] | ETR  [95% CI] | p | Ratio to baseline [SE] |
| **Lipid endpoints** | | | | | | | | |
| Free fatty acids, mmol/l | 0.6  [54.33] | 1.2  [0.04] | 0.90  [0.81; 1.01] | 0.0661 | 1.2  [0.05] | 0.94  [0.85; 1.05] | 0.3082 | 1.3  [0.05] |
| Total cholesterol, mg/dl | 177.0  [24.47] | 0.9  [0.01] | 0.95  [0.91; 0.99] | 0.0146 | 0.9  [0.01] | 0.97  [0.93; 1.02] | 0.2174 | 1.0  [0.02] |
| HDL cholesterol, mg/dl | 46.6  [30.35] | 1.0  [0.01] | 0.99  [0.95; 1.03] | 0.7298 | 1.0  [0.01] | 1.01  [0.97; 1.05] | 0.7268 | 1.0  [0.01] |
| LDL cholesterol, mg/dl | 94.1  [40.87] | 0.9  [0.03] | 0.93  [0.84; 1.03] | 0.1444 | 0.9  [0.03] | 0.98  [0.89; 1.08] | 0.7173 | 0.9  [0.03] |
| VLDL, mg/dl | 27.7  [59.73] | 1.0  [0.03] | 0.91  [0.82; 1.01] | 0.0720 | 1.0  [0.03] | 0.90  [0.81; 0.99] | 0.0354 | 1.1  [0.04] |
| Triglycerides, mg/dl | 142.9  [64.02] | 1.0  [0.03] | 0.92  [0.83; 1.02] | 0.1223 | 1.0  [0.03] | 0.90  [0.81; 1.00] | 0.0476 | 1.1  [0.04] |

CV, coefficient of variation; ETR, estimated treatment ratio to placebo; HDL, high-density lipoprotein; LDL, low-density lipoprotein; VLDL, very low-density lipoprotein.

## Table S5. Hypoglycemic episodes

|  | | | **Semaglutide 0.5 mg** | | | | **Semaglutide 1.0 mg** | | | | **Placebo** | | | |
| --- | --- | --- | --- | --- | --- | --- | --- | --- | --- | --- | --- | --- | --- | --- |
|  | | | N | (%) | E | R | N | (%) | E | R | N | (%) | E | R |
| N | | | 132 |  |  |  | 131 |  |  |  | 133 |  |  |  |
| Severe or blood glucose-confirmed symptomatic^a^  Nocturnal severe or blood glucose-confirmed symptomatic^a^ | | | 11  2 | (8.3)  (1.5) | 17  3 | 20.2  3.6 | 14  4 | (10.7)  (3.1) | 25  6 | 30.5  7.3 | 7  4 | (5.3)  (3.0) | 13  6 | 15.5  7.1 |
| Severe  Nocturnal severe | | | 0  0 |  |  |  | 2  0 | (1.5) | 2 |  | 1  0 | (0.8) | 1 |  |
|  |  | **In subjects with HbA_1c_ ≤8% at screening** | | | | | | | | | | | |  |
| N | | | 49 |  |  |  | 49 |  |  |  | 49 |  |  |  |
| Severe or blood glucose-confirmed symptomatic | | | 4 | (8.2) | 5 | 15.7 | 8 | (16.3) | 14 | 46.5 | 2 | (4.1) | 3 | 9.9 |
|  |  | **In subjects with HbA_1c_ >8%** | | | | | | | | | | | |  |
| N | | | 83 |  |  |  | 82 |  |  |  | 84 |  |  |  |
| Severe or blood glucose-confirmed symptomatic | | | 7 | (8.4) | 12 | 22.9 | 6 | (7.3) | 11 | 21.2 | 5 | (6.0) | 10 | 18.6 |

Severe or blood-glucose-confirmed symptomatic hypoglycemia: an episode that is severe according to the ADA classification or blood glucose-confirmed by a plasma glucose value below 3.1 mmol/l (56 mg/dl) with symptoms consistent with hypoglycemia.

^a^The estimated odds for experiencing severe or blood glucose-confirmed episodes did not differ statistically significantly with both doses of semaglutide when compared with placebo. E, number of events; HbA_1c_, glycated hemoglobin; N, number of subjects; R, events per 100 patient years of exposure.

## Table S6. Amylase and lipase at Week 30 by treatment group

|  | **Semaglutide 0.5 mg**  **(n=132)** | | | | **Semaglutide 1.0 mg**  **(n=131)** | | | | **Placebo**  **(n=133)** | |
| --- | --- | --- | --- | --- | --- | --- | --- | --- | --- | --- |
|  | Mean at Week 30 [SE] | Ratio to baseline [SE] | ETR  [95% CI] | p | Mean at Week 30 | Ratio to baseline [SE] | ETR  [95% CI] | p | Mean at Week 30 | Ratio to baseline [SE] |
| Amylase (U/l) | 61.9  [1.39] | 1.1  [0.02] | 1.15  [1.08; 1.22] | <0.0001 | 63.4 | 1.1  [0.03] | 1.18 [1.11; 1.26] | <0.0001 | 53.8 | 1.0  [0.02] |
| Lipase (U/l) | 49.3  [1.97] | 1.3  [0.05] | 1.35  [1.21; 1.51] | <0.0001 | 46.6  [1.88] | 1.2  [0.05] | 1.28  [1.14; 1.43] | <0.0001 | 36.5 | 1.0  [0.04] |

ETR, estimated treatment ratio to placebo.
